# Metal–ligand covalency enables room temperature molecular qubit candidates†
†Electronic supplementary information (ESI) available: Methods and additional characterization and discussion. Crystallographic information of **1**, **3–8** can be obtained from the Cambridge Structural Database CCDC 1877212–1877218. For ESI and crystallographic data in CIF or other electronic format see DOI: 10.1039/c9sc00074g


**DOI:** 10.1039/c9sc00074g

**Published:** 2019-05-31

**Authors:** Majed S. Fataftah, Matthew D. Krzyaniak, Bess Vlaisavljevich, Michael R. Wasielewski, Joseph M. Zadrozny, Danna E. Freedman

**Affiliations:** a Department of Chemistry , Northwestern University , Evanston , IL 60208 , USA . Email: m-wasielewski@northwestern.edu ; Email: danna.freedman@northwestern.edu; b The Institute for Sustainability and Energy at Northwestern , Northwestern University , Evanston , IL 60208 , USA; c Department of Chemistry , University of South Dakota , Vermillion , South Dakota 57069 , USA; d Department of Chemistry , Colorado State University , Fort Collins , Colorado 80523 , USA . Email: joe.zadrozny@colostate.edu

## Abstract

Harnessing synthetic chemistry to design electronic spin-based qubits, the smallest unit of a quantum information system, enables us to probe fundamental questions regarding spin relaxation dynamics. We sought to probe the influence of metal–ligand covalency on spin–lattice relaxation, which comprises the upper limit of coherence time. Specifically, we studied the impact of the first coordination sphere on spin–lattice relaxation through a series of four molecules featuring V–S, V–Se, Cu–S, and Cu–Se bonds, the Ph_4_P^+^ salts of the complexes [V(C_6_H_4_S_2_)_3_]^2–^ (**1**), [Cu(C_6_H_4_S_2_)_2_]^2–^ (**2**), [V(C_6_H_4_Se_2_)_3_]^2–^ (**3**), and [Cu(C_6_H_4_Se_2_)_2_]^2–^ (**4**). The combined results of pulse electron paramagnetic resonance spectroscopy and ac magnetic susceptibility studies demonstrate the influence of greater M–L covalency, and consequently spin-delocalization onto the ligand, on elongating spin–lattice relaxation times. Notably, we observe the longest spin–lattice relaxation times in **2**, and spin echos that survive until room temperature in both copper complexes (**2** and **4**).

## Abstract 2

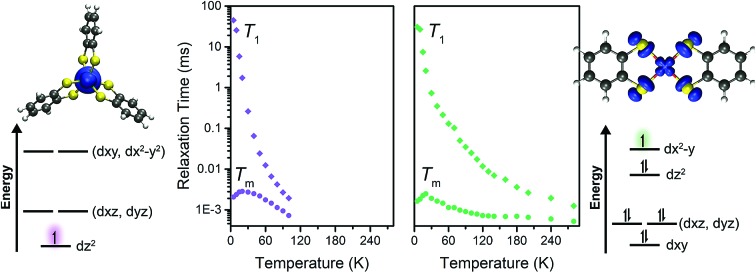
Metal–ligand covalency enables observation of coherent spin dynamics to room temperature in a series of vanadium(iv) and copper(ii) catechol complexes.

## Introduction

Magnetic transition metal complexes are a promising platform to create qubits, the smallest unit of quantum information science (QIS) systems. Within the broad scope of QIS there are numerous applications, most prominently, quantum computing which offers the potential to revolutionize our approach to certain computational problems.^1^–^3^ A second emerging area of QIS, well suited to molecular intervention, is quantum sensing, wherein quantum objects are used as environmental probes.^4^ Magnetic complexes comprise a highly promising platform to develop design principles for the foregoing applications owing to their wide range of tunability.^5^–^7^

To develop systems for QIS, it is essential to design qubits with long spin–spin and spin–lattice relaxation times, *T*_2_ and *T*_1_, respectively. In quantum computing, *T*_2_ represents the lifetime of information, while, *T*_1_ signals the maximum memory storage time, as well as the minimum possible time length between each computational cycle. For applications within quantum sensing, it is possible harness the sensitivity of the timescales of *T*_1_ and *T*_2_ to the local chemical and magnetic environment of the electronic spin to probe the local chemical environment. Both of these applications rely on an explicit understanding of how molecular factors influence *T*_1_ and *T*_2_ and is of intense current interest.^8^–^12^ Owing to the inherent chemical tunability of spins in transition metal complexes, investigating *T*_1_ and *T*_2_ in such species is a promising route to the requisite knowledge.

By modifying the chemical structure of molecules, it is possible to tune the specific lattice vibrations thereby engendering changes to *T*_1_. Prior research into molecular design of candidate qubits focused on lengthening *T*_1_ and *T*_2_ and increasing their persistence to higher temperatures. Specifically, the role phonons and local vibrational modes play in modulating spin dynamics of electronic spin qubits is of particular importance.^13^–^19^ In this study, we focus on a different parameter, modulating the covalency of the metal–ligand bond to control relaxation times. The identity of the spin-bearing orbital and its interaction with the lattice and intramolecular vibrations should guide the magnitude of *T*_1_. A nonbonding orbital, for example, should interact less strongly with lattice vibrations than a bonding (or antibonding) orbital, and thus display a longer *T*_1_. Within this framework, we hypothesize that tuning the covalency of metal–ligand bonding could likewise enhance *T*_1_. Despite this intuitive picture, significant experimental work remains to be done to test its validity.

Similarly, metal–ligand covalency could be envisioned to impact *T*_2_ by modulating the interaction of an electronic spin with nearby nuclear and electronic spins.^20^ When these environmental spins undergo flip-flop motions, such interactions shorten *T*_2_. Here, delocalization of the metal-based spin toward the ligand-based nuclear spins may engender stronger magnetic interactions that shut down flip-flop motions and lead to longer *T*_2_ times. Metal ions ligated by nuclear spin-free ligand shells completely eliminate such flip-flops, which enable near-millisecond-length *T*_2_ times.^21^,^22^ Yet that design principle is chemically limited, and hence, methods of enabling nuclear spins to be incorporated into the synthetic design are important.

**Fig. 1:**
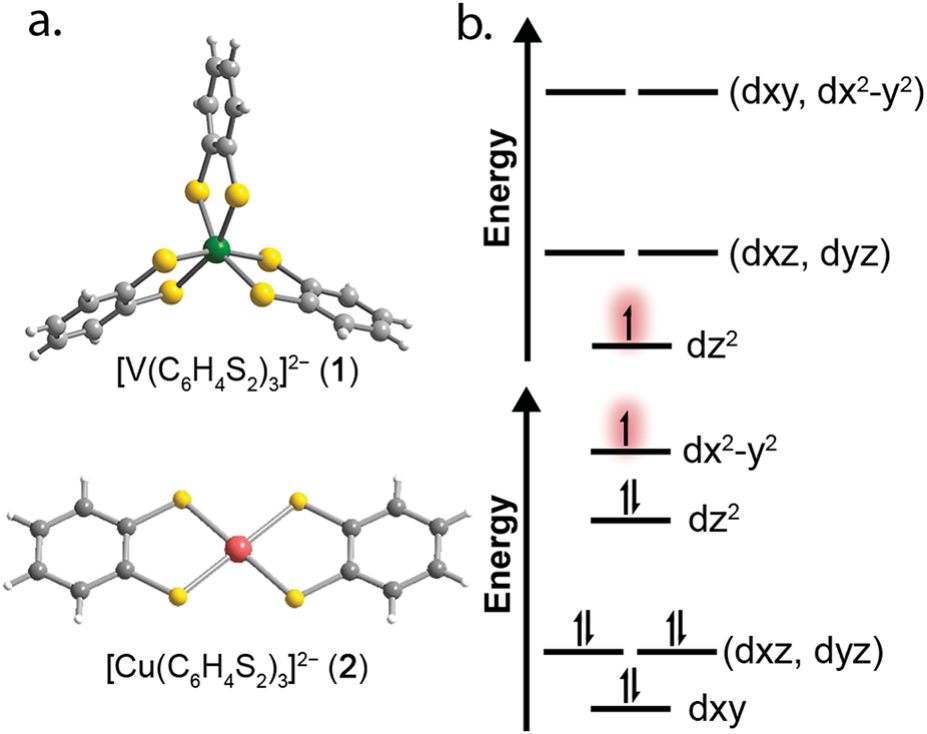
(a) Molecular structures of the [VL_3_]^2–^ and [CuL_2_]^2–^ units as determined in the crystal structures of **1** and **2**. Dark green, red, yellow, gray, and white spheres represent vanadium, copper, sulfur, carbon, and hydrogen atoms, respectively. (b) Qualitative d-orbital splitting diagrams for **1** (top), and **2** (bottom) highlight the nature of the orbital singly occupied by an unpaired spin.

To test this hypothesis we synthesized and investigated the Ph_4_P^+^ salts of the dithiocatecholate complexes of vanadium(iv) and copper(ii):^23^,^24^ [V(C_6_H_4_S_2_)_3_]^2–^ (**1**), [Cu(C_6_H_4_S_2_)_2_]^2–^ (**2**), and the diselenocatecholate complexes [V(C_6_H_4_Se_2_)_3_]^2–^ (**3**), and [Cu(C_6_H_4_Se_2_)_2_]^2–^ (**4**) (Fig. 1). Here, we hypothesized that direct comparison of the V(iv) and Cu(ii) complexes would offer insight into the impact of changing the nature of the molecular orbital in which the electronic spin resides; a non-bonding, low-covalency d_*z*^2^_ orbital and an anti-bonding, high-covalency d_*x*^2^–*y*^2^_ orbital, respectively. Notably, in the V(iv) complex (**1**) the echo disappears beyond 100 K, whereas in the Cu(ii) complex (**2**) the spin echo persists until room temperature. We attribute this difference in behavior to a change in metal–ligand covalency. Investigation of **3** and **4** in concert with **1** and **2** allowed us to systematically test the role that more diffuse, 4p donor atoms play in modulating covalency and, thus, spin relaxation times. Note, because all complexes feature ligands that contain proton nuclear spins (^1^H, *I* = 1/2, 99.98% natural abundance) at nearly identical distances from the spin-bearing metal ion, the series provides a qualitative picture of the role of the variation in metal–ligand covalency on *T*_2_ times with a constant number of nuclear hyperfine interactions.

## Results and discussion

Correlating spin–lattice relaxation with structural changes necessitates a clear understanding of the structural similarities and differences between the compounds. Notably, the two classes of complexes have significantly different structures. The V^4+^ complex (**1**) is a hexacoordinate tris-chelate complex in a pseudo-octahedral geometry, while in **2**, the Cu^2+^ ion resides in a four-coordinate, square planar geometry. In the solid state, the structure of **1** deviates from an idealized trigonal geometry owing to distortions in the arrangements of ligands. The average bond distances between the metal ions and the ligand donor atoms, 2.372(11) Å and 2.279(2) Å for V–S and Cu–S bonds, respectively, agree well with prior crystal structures of other dithiolate species.^25^ Comparison of the average M–L bond distances in **1** and **2** reveals that the metal–donor bond distances are all well below those computed using the Shannon–Prewitt ionic radii for Cu^2+^, V^4+^, and S^2–^, with larger deviations observed for **2** relative to **1**.^26^ These short bond distances suggest enhanced metal–donor covalency in the copper complex relative to its vanadium counterpart. To further probe M–L covalency we investigated both complexes *via* X-band (∼9.5 GHz) continuous wave (cw) and pulse electron paramagnetic resonance (EPR) spectroscopy. As interactions between separate magnetic molecules engender spin–spin relaxation, we synthesized their closed-shell analogues: (Ph_4_P)_2_[Ti(C_6_H_4_S_2_)_3_] and (Ph_4_P)_2_[Ni(C_6_H_4_S_2_)_2_]. We then diluted complexes **1** and **2** in a matrix of their respective diamagnetic analogues at concentrations of 0.5%, yielding the compounds (Ph_4_P)_2_[V_0.005_Ti_0.995_(C_6_H_4_S_2_)_3_] (**1′**) and (Ph_4_P)_2_[Cu_0.005_Ni_0.995_(C_6_H_4_S_2_)_2_] (**2′**) (see ESI†).

**Fig. 2:**
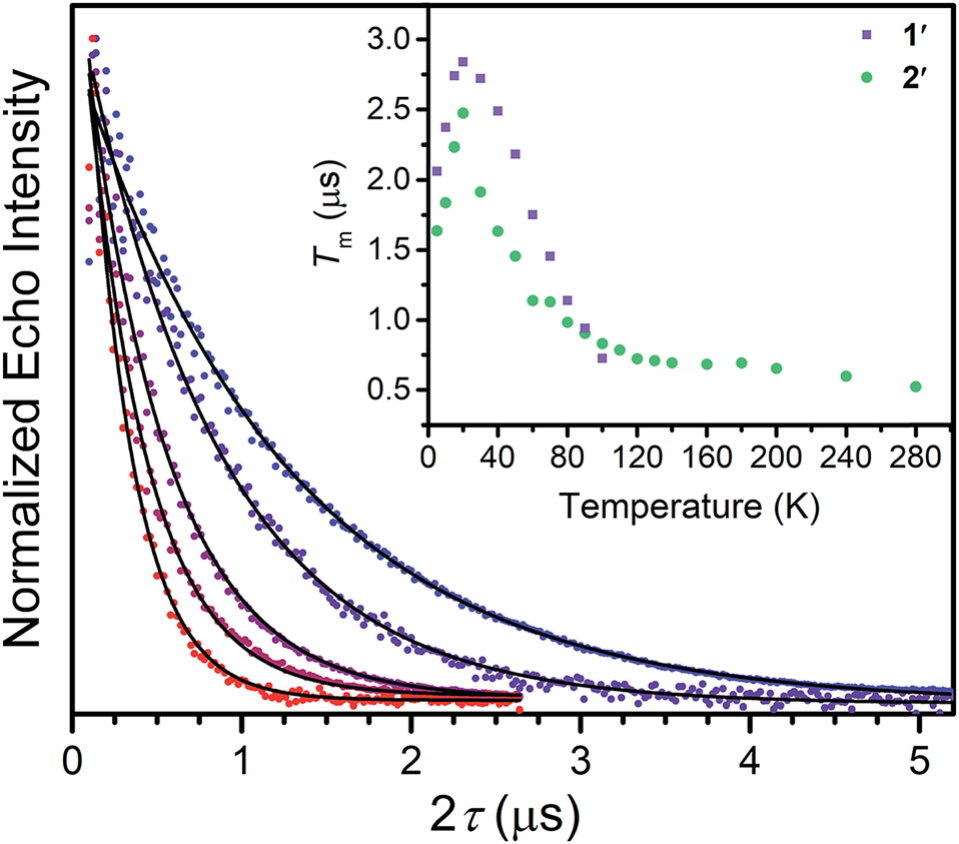
Overlay of select *T*_m_ decay curves, in the temperature range of 20–280 K. The data was collected through application of a Hahn-echo pulse sequence. Inset: Temperature dependence of electronic spin phase memory times (*T*_m_) for **1′** and **2′**. Errors in the data are smaller than the dimensions of the data points.

We investigated the diluted compounds by pulse EPR spectroscopy to probe the impact of covalency on spin dynamics. A direct measurement of *T*_2_ is often not possible. Instead the phase memory time, *T*_m_, which encompasses all processes that contribute to electron spin decoherence, which include the *T*_2_ of the electron spin, is measured. Measurement of *T*_m_ proceeded *via* application of a Hahn-echo pulse sequence to **1′** and **2′** in the temperature range of 5–280 K (Fig. 2). At 5 K, both complexes feature *T*_m_ values of 1.5–2 μs, within the typical range for transition metal complexes. With increasing temperature from 5 K, *T*_2_ increases by ∼1 μs in both complexes. This lengthening occurs until 20 K, wherein *T*_m_ peaks for **1′** and **2′**, reaching values of 2.84(1) and 2.48(2) μs, respectively. The origin of this behavior remains unclear but has been observed in other V(iv) catecholate complexes.^19^ Above 20 K, *T*_2_ begins to decrease, whereby **1′** features a more dramatic temperature dependence relative to **2′**. By 100 K, **1′** possesses a *T*_m_ value of 0.72(6) μs, only slightly shorter than the *T*_m_ value of 0.83(1) μs for **2′** at 100 K. Interestingly, the echo is no longer detectible in **1′** above 100 K. In contrast, **2′** displays an echo until 280 K, permitting measurement of *T*_m_ at room temperature, with *T*_m_ = 0.51(1) μs at 280 K. The drastic discrepancies observed between **1′** and **2′**, as well as the marginal decrease in *T*_m_ across such a wide temperature range in **2′** prompted us to delve deeper into their electronic structures and evaluate the impact of M–L covalency on *T*_1_ to account for the observed temperature dependences.

**Fig. 3:**
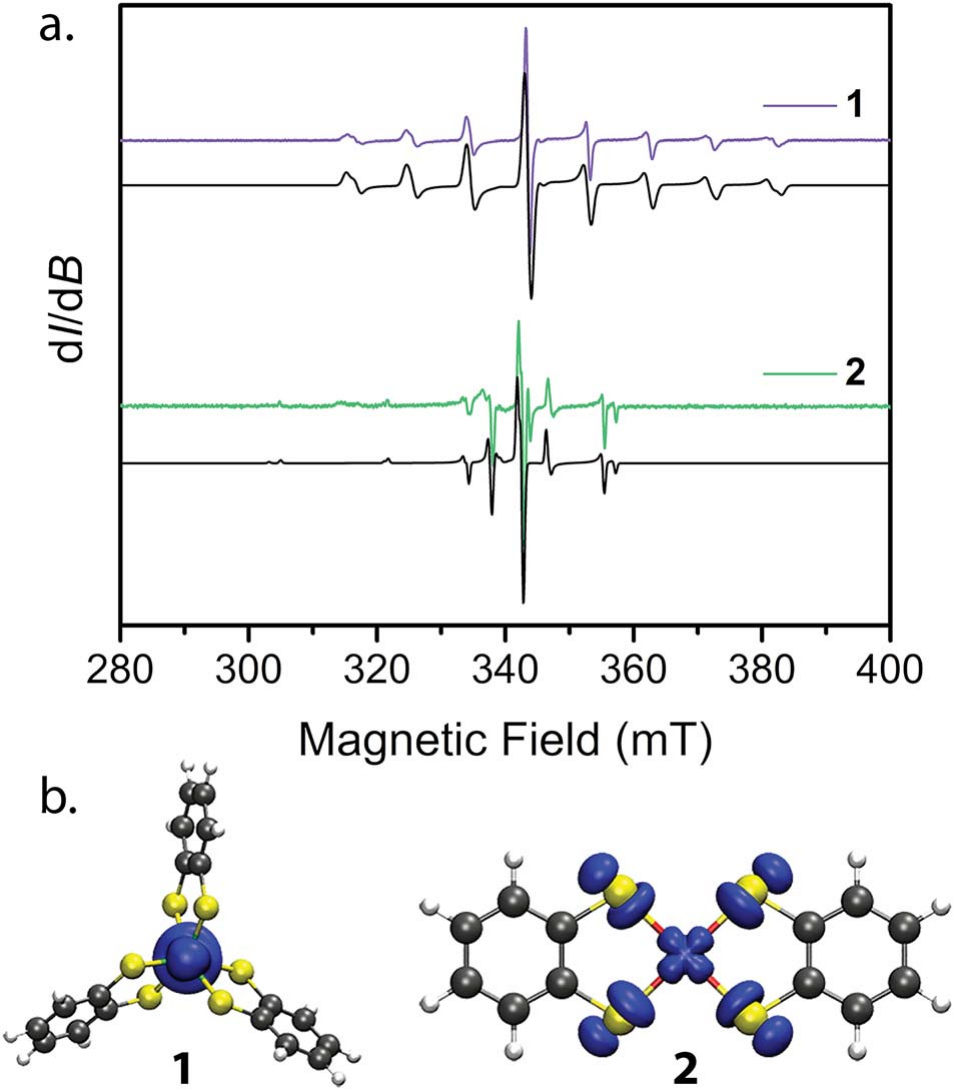
(a) cw EPR spectra collected at 298 K for **1′** and **2′** (colored spectra) and their best simulations (black). Spin Hamiltonian parameters responsible for the simulations are reported in Table 2. (b) Plots of spin densities from the M06-L calculation for **1** and **2** with DMF as the solvent. The plots highlight the degree of spin delocalization onto the S-atoms in **2** and lack thereof in **1**.

Towards that end, we examined the diselenocatecholate analogues of **1′** and **2′**: (Ph_4_P)_2_[V_0.005_Ti_0.995_(C_6_H_4_Se_2_)_3_] (**3′**) and (Ph_4_P)_2_[Cu_0.005_Ni_0.995_(C_6_H_4_Se_2_)_2_] (**4′**) (see ESI† for full details). We initiated our investigation by analysis of their respective cw EPR spectra. **1′** and **3′** exhibit a collection of eight lines over 100 mT, consistent with electron–nuclear hyperfine coupling between the *S* = 1/2 spins and the 100% naturally abundant *I* = 7/2 ^51^V nuclei (Fig. 3a). Complexes **2′** and **4′** display a more complex collection of resonances which are attributed to hyperfine coupling between the axial *S* = 1/2 spin with the two naturally abundant *I* = 3/2 nuclear spins of copper (^63^Cu and ^65^Cu, 69% and 31% natural abundance, respectively). Additional peaks in the spectrum of **4′** likely result from coupling to the Se–donor atoms (^77^Se, *I* = 1/2, 7.6% natural abundance, see ESI Fig. S5†). Simulations of all spectra proceeded with the program Easyspin^27^ and the spin Hamiltonian:

1 *Ĥ* = ***g**μ*_B_***BS*** + ***IAS***

where ***g*** is the rhombic *g*-factor, *μ*_B_ the Bohr magneton, ***B*** the magnetic field, ***S*** the electronic spin, ***I*** the nuclear spin of the metal nucleus, and ***A*** the rhombic hyperfine coupling. Best simulations of the spectra for each complex yielded values that are reported in Table 1. The *A* and *g* parameters for **1′** and **3′** are within the expected values for trigonally symmetric pseudo-octahedral vanadium complexes.^12^,^21^ A cw EPR spectrum of **2** was previously reported,^24^ and the reported parameters reasonably reproduce our spectra with the inclusion of hyperfine coupling to both ^63^Cu and ^65^Cu nuclei. The additional complexity of the cw spectrum of **4′** is well-modelled with the inclusion of hyperfine coupling to a 7.6% natural abundance of ^77^Se nuclei. However, the simulation of **4′** is not a precise match, which may stem from non-collinearity of ***A*** and ***g***, which has been previously reported in copper bis-diselenoate complexes.^28^

**Table 1:** Spin Hamiltonian parameters for simulating the cw EPR spectra of **1′–4′**

	**1′**	**2′**	**3′**	**4′** ^*a*^
*g*_∥_	1.9878	2.085	1.950	2.082
*g*_⊥_ ^*b*^	1.9698	2.019	1.960	2.018
	1.9698	2.019	1.955	2.018
*A*_∥_ (MHz)	0^*c*^	500	0^*c*^	460 (140)
*A*_⊥_ (MHz)^*b*^	258	115	255	145 (90)
	264	115	265	145 (90)
*α* ^2^	—	0.51	—	0.39
Spin density (*M*)	0.935	0.756	0.949	0.732
Spin density (*E*)^*d*^	0.008	0.059	0.007	0.065

^*a*^Hyperfine coupling constants in parentheses for **4′** correspond to ^77^Se hyperfine coupling.

^*b*^Top values for *g*_⊥_ and *A*_⊥_ are *g*_*x*_ and *A*_*x*_, bottom values are *g*_*y*_ and *A*_*y*_.

^*c*^No features in the EPR spectrum corresponding to *A*_∥_ are apparent, inducing significant error in this value, and was held at zero for the simulation.

^*d*^Spin densities at the metal (*M*) and S/Se donors (*E*) were calculated using CASPT2.

**Table 2:** Fit parameters to the temperature dependence of *T*_1_ for **1′–4′**

	**1′**	**2′**	**3′**	**4′**
*A*_Dir_ (ms^–1^ K^–1^)	4.7 ± 0.4	4.8 ± 0.8	0.51 ± 0.3	2.25 ± 0.8
*B*_Ram_ (ms^–1^)	5 ± 2 × 10^5^	1.4 ± 0.4 × 10^5^	5.1 ± 2 × 10^5^	2.1 ± 1 × 10^6^
*C*_Loc_ (ms^–1^)	5.4 ± 3 × 10^6^	2.6 ± 1 × 10^6^	3.1 ± 2 × 10^6^	3.1 ± 2 × 10^6^
*Θ*_Deb_ (K)	98 ± 15	94.9 ± 9	71 ± 20	89 ± 6
*Δ*_loc_ (cm^–1^)	275 ± 40	488 ± 72	161 ± 51	343.9 ± 80

The continuous wave EPR spectral simulations provide insight into the M–L covalency of **1–4**. The magnitude of *A* is an important proxy of spin density at the metal nucleus, which is modified by covalency between the metal and ligands. This covalency is significantly dependent on the spin-bearing orbital. Only a small change is observed in *A* by changing the donor atom from S to Se in **1′** and **3′**. Meanwhile, the same variation of donor atom in **2′** and **4′** results in a significant decrease in the magnitude and axiality of *A*. Of additional importance is the decrease in magnitude of *A* from **2′** to **4′**; whereby the weaker hyperfine interaction in **4′** indicates relatively greater spin delocalization onto the C_6_H_4_Se_2_^2–^ ligand compared to C_6_H_4_S_2_^2–^. To further bolster this argument, extraction of the covalency parameter *α*^2^, which relates to the σ bond strength of the d_*x*^2^–*y*^2^_ orbital, from the *A* and *g* parameters provides insight into the degree of spin-delocalization onto the catecholate ligands.^29^,^30^ This analysis, commonly performed in square planar Cu(ii) complexes,^31^–^33^ yields *α*^2^ parameters of 0.51 and 0.39 for **2′** and **4′**, respectively. Here, a smaller *α*^2^ supports a greater degree of spin-delocalization onto the selenocatecholate ligand in **4′** relative to its sulfur analogue in **2′**.

We employed both CASSCF/CASPT2 and DFT calculations on **1–4** to calculate the *g*-tensors and determine the amount of spin-density on the metal centers across the series. The calculated *g*-tensors agree well with the values extracted from the simulations of the cw spectra (see ESI Table S26†). The results of the MS-CASPT2 calculations, presented in Table 1, reveal significant spin densities residing on the S/Se donor atoms in **2** and **4**, respectively. This showcases the strong covalency between the Cu^2+^ d_*x*^2^–*y*^2^_ orbital and S 3p and Se 4p orbitals. This result is in stark contrast with their vanadium counterparts, where spin density resides primarily in the d_*z*^2^_ orbital of the vanadium centre (Fig. 3b). The calculations are in good agreement with the natural bond orbital analysis computed with the M06-L functional and corroborate the *α*^2^ analysis extracted from the cw EPR spectra (see ESI Fig. S22 and Table S25†). The aggregate of this data support larger spin densities at the Se relative to S donors in **4** and **2**, respectively. These results are broadly consistent with the qualitative electronic structures of Fig. 1:^34^ a (d_*z*^2^_)^1^ electron configuration in **1** and **3**, wherein the d_*z*^2^_ orbital is relatively nonbonding, and a (d_*x*^2^–*y*^2^_)^1^ configuration in **2** and **4**, wherein the spin-bearing d_*x*^2^–*y*^2^_ orbital directly engages the ligand orbitals. In summary, these data suggest: (1) enhanced M–L covalency for the copper-containing complexes **2** and **4** relative to **1** and **3**; and (2) greater M–L covalency with the C_6_H_4_Se_2_^2–^ ligand relative to the C_6_H_4_S_2_^2–^ ligand.

**Fig. 4:**
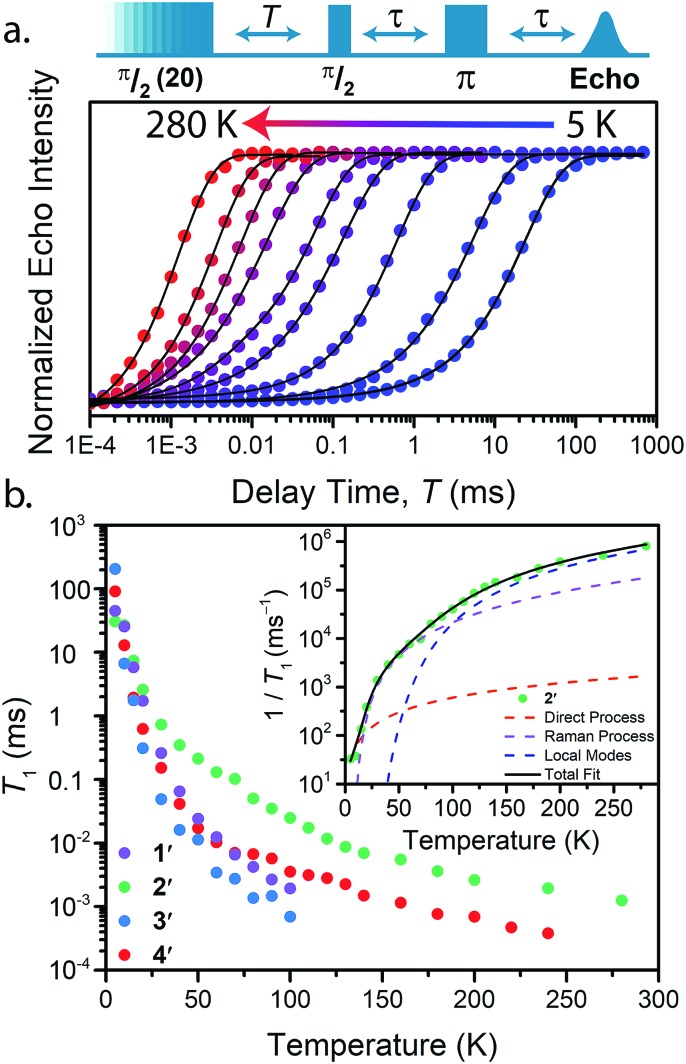
(a) Select variable temperature saturation recovery curves for **3′**. The data were collected *via* application of the pulse sequence depicted above the plot, in the temperature range of 5 to 280 K. (b) Summary of temperature dependent *T*_1_ data determined for **1′–4′**. Inset: Fit to the temperature dependence of *T*_1_ for **2′**, highlighting the relaxation mechanisms discussed in the text.

With these aspects of their electronic structures established, we explored the potential impact(s) on the spin–lattice relaxation times *via* saturation recovery experiments (Fig. 4a). In this experiment, a train of twenty consecutive 12 ns microwave pulses are applied to saturate the spin resonance corresponding to the *M*_*S*_ = –1/2 to *M*_*S*_ = +1/2 transition. Following saturation, a two-pulse Hahn-echo sequence is applied to detect the resurrection of the signal as a function of delay time, *T*. This pulse sequence differentiates itself from the commonly utilized inversion recovery experiment in that it seeks to eliminate the influence of spectral diffusion, or cross relaxation, which is often a prominent relaxation mechanism accounting for deviations from single exponential decay dynamics at low temperatures.^35^ Plotting the magnitude of the echo intensity as a function of delay time on a logarithmic scale results in the sigmoidal curves presented in Fig. 4a. Fitting these recovery curves to exponential decay functions, modified for the inclusion of spectral diffusion (see ESI†), provided the rates of recovery, which are equal to 1/*T*_1_.^36^ For all complexes, saturation recovery is slowest at 5 K and becomes faster with increasing temperature. In **1′** and **3′**, the echo is detectable up to 100 K, beyond which the relaxation times became too fast for observation. In **2′** and **4′**, however, a detectable echo persists until 280 K. The qualitative observation of slower relaxation at low temperature relative to high temperature is here quantitated. At 5 K, *T*_1_ values for **1′–4′** are 25(1), 30(1), 205(9), and 91(2) ms, respectively, while at 100 K, *T*_1_ is exponentially shorter, 1.9(1), 24.8(10), 0.7(1), and 3.5(1) μs for **1′–4′**, respectively.

Analysis of the temperature dependence of *T*_1_ reveals three relaxation mechanisms that govern *T*_1_ for **1′–4′**. The temperature dependence of *T*_1_ for all complexes was modeled to account for the influence of the direct, Raman, and local modes (Fig. 4b inset), using eqn (1) below.^37^2
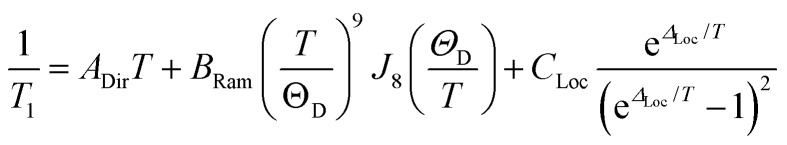

*A*_Dir_, *B*_Ram_, and *C*_Loc_ are the coefficients for the direct, Raman, and local modes, respectively. *T* is the temperature, *Θ*_D_ is the Debye temperature, *J*_8_ is the transport integral, and *Δ*_Loc_ is the energy of the operative local vibrational mode. Our assignment of these processes, which models the data quite well, is guided by the known temperature ranges in which they occur. First, the direct process, which proceeds by the emission of a phonon is dominant below 10 K, and imparts a linear temperature dependence on *T*_1_. The direct process and spectral diffusion are often convoluted at these low-temperatures, but the use of a saturation recovery pulse sequence eliminates spectral diffusion from our model. Second, from 10 to 80 K, the Raman mechanism is typically observed, a two-phonon process involving the simultaneous absorption and emission of a phonon and imparts an exponential temperature dependence on *T*_1_.^38^ At higher temperatures, the influence of local vibrational modes begins to take effect, prompting our use of the local-mode term. This last term acts by modulating the *M*_*S*_ energy levels, and is likely dominated by the first coordination sphere around the metal center.^13^ We eliminated a common high-temperature process, the Orbach process, from our model owing to the lack of low-lying accessible electronic states in dilute, uncoupled *S* = 1/2 systems.

The results of the foregoing mechanistic analyses of **1′–4′** point toward a picture of high-temperature relaxation governed primarily by metal–ligand interactions. First, the Raman process and local modes are considerably less operative (evidenced by smaller *B*_Ram_) in the thiocatechol complexes **1′** and **2′***versus* their selenocatechol analogues **3′** and **4′**. Second, the values of *Δ*_Loc_ extracted from the fits are lower for the V(iv) complexes (275 and 161 cm^–1^ for **1′** and **3′**) than the Cu(ii) complexes (488 and 343 cm^–1^ for **2′** and **4′**, respectively). These observations are consistent with differences in metal–ligand interactions in **1–4**. For example, the magnitude of *B*_Ram_ is strongly dependent on spin–orbit coupling and is concomitant with the observed differences in transitioning from S– (**1** and **2**) to their heavier Se–donor analogues (**3** and **4**).^39^ However, a more pronounced increase in *B*_Ram_ is observed between **2** and **4** relative to **1** and **3**. Separately, *Δ*_Loc_ is dependent on intramolecular vibration energies, and likely dominated by the inner coordination sphere and metal–ligand bond strength. The diminished influence of the local modes in **2** and **4** accounts for the persistence of *T*_1_ until room temperature.^18^

Our *T*_1_ analysis led us to believe that van der Waals phonon modes dominate *T*_1_ below 100 K and local modes of vibration dominate the *T*_1_ dynamics above 100 K. To support our analysis, we computed the phonon density of states and the local vibrational modes for **1–4**, the results of which can be found in the ESI (Fig. S25 and S26†). Below 100 K, the Raman process dominates *T*_1_ relaxation and proceeds *via* coupling to low-energy phonon modes. The phonon density of states supports our observed trend in *T*_1_ relaxation below 100 K (Fig. S26†). Specifically, **3** and **4** possess a larger density of low-energy (<200 cm^–1^) phonon states compared to **2**, and accounts for the longer *T*_1_ values for **2** in the 20–100 K range (see ESI† for further discussion). Likewise, the vanadium complexes **1** and **3** possess a significantly larger number of low-energy local vibrational modes relative to their copper counterparts. Additionally, the selenium–donor complexes **3** and **4** possess lower energy local vibrational modes in comparison to their sulfur–donor analogues. These observations corroborate the trend in *Δ*_Loc_ extracted from our *T*_1_ analysis. We believe the higher energy vibrational modes in **2** relative to **4** accounts for the longer *T*_1_ in **2** at temperatures above 100 K.

**Fig. 5:**
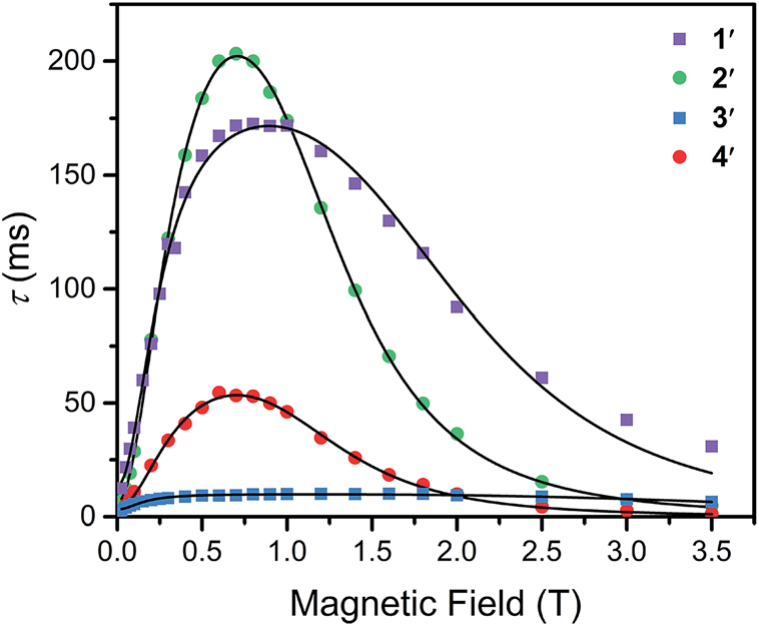
Variable-field relaxation times (*τ*) extracted from alternating current magnetic susceptibility measurements performed on **1–4** at 5 K between 0.025 and 3.5 T. The solid lines are the best fits to the data according to eqn (3) in the text.

To further probe the impact of metal–ligand covalency on spin–spin interactions at low temperatures we acquired variable-field alternating current (ac) magnetic susceptibility data on **1–4** at 5 K from 25 mT to 3.5 T. We employed this technique to provide further insight into the spin relaxation dynamics operative at the lowest temperatures of measurement, specifically cross-relaxation and the direct process (Fig. 5 and S10–S13). Here, a peak in the out-of-phase magnetic susceptibility at a given oscillating field, frequency, static magnetic field, and temperature yields the rate of spin–lattice relaxation, 1/*τ*. This variable-field technique reveals additional mechanistic information that variable-temperature measurements does not.^40^–^43^ Close examination of the variable-field measurements performed at 5 K reveals a qualitatively similar field-dependence of *τ* for each compound. At low magnetic fields, *τ* increases with increasing magnetic fields until ∼0.75 T, beyond which it begins to drastically decrease with increasing magnetic fields. We modelled this data with the Brons–van Vleck model^44^ (eqn (2)) (see Table S23† for fit parameters):3
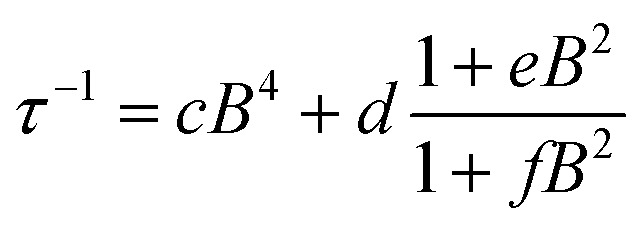
here, *c* is the coefficient for the direct process, *d* is the zero-field relaxation rate, *e* is dictated by the spin-concentration, and *f* relates to the internal magnetic field generated by dipolar coupled spins. This final parameter quantitates the ability of the external magnetic field to suppress cross-relaxation.

The Brons–van-Vleck model fits the data for **1–4** quite well and provides a mechanistic description of the variable-field data. First, the onset of the direct process at higher magnetic fields is responsible for the high-field hastening of relaxation, as it exhibits a *B*^4^ dependence owing to availability of a larger density of phonon states.^45^ Second, the low magnetic field behavior stems from shutting down dipolar induced (*e.g.* electron–electron and electron–nuclear) cross-relaxation pathways, leading to an increase in *τ*. This phenomenon is also observed in nitrogen vacancy centers in diamond.^46^ Finally, the plateau observed at ∼0.75 T in all compounds arises from the competition between subduing cross-relaxation at low fields and enabling the direct process at high fields.^37^

The more prominent decrease in *τ* at high-fields for **2** and **4**, parameterized by *c*, are quantitated to be an order of magnitude more susceptible to the direct process relative to **1** and **3** (see ESI Table S23†). We hypothesize this behavior arises from stronger electron–nuclear hyperfine coupling in **2** and **4** relative to their vanadium counterparts, as mixing of magnetic sublevels enables the direct process.^47^,^48^ This will strongly affect the spin–lattice relaxation times at the lowest temperatures of measurement, and more critically at higher magnetic fields.

The ability of the external magnetic field to suppress cross-relaxation in these species relates to the internal magnetic field generated by the surrounding spin bath. We find *f* is considerably smaller in the vanadium complexes (212(62) and 122(23) T^–2^ for **1** and **3**, respectively) relative to the copper complexes (389(50) and 162(30) T^–2^ for **2** and **4**, respectively). The parameter *f* inversely scales with the number of nuclear spins that are coupled to electron spins by the hyperfine and dipolar interaction. For example, *f* is markedly smallest in **3**, where the vanadium(iv) ion is expected to experience dipolar coupling to twelve catecholate ^1^H spins and six ^77^Se nuclei. Therefore, **3** exhibits the most pathways to undergo cross-relaxation. The more severe reduction in *f* between **2** and **4** relative to **1** and **3** is the result of the stronger ^77^Se hyperfine interaction arising from the strong Cu^2+^–Se covalency, thereby hindering the ability of the field to suppress cross-relaxation involving the ^77^Se nuclear spins. Our analysis corroborates the idea that hyperfine interactions enable the direct process to dominate at high magnetic fields and enable cross-relaxation pathways at low magnetic fields. Indeed, a recent theoretical study highlights the influence of hyperfine interactions on accelerating spin-relaxation, demonstrating its dominant influence at low magnetic fields.^49^ In summary, these results inspire the design principle of eliminating nuclear spins from ligands of qubit candidates due to their detrimental impact on both *T*_1_ and *T*_2_.^50^

The aggregate of our data thus far permits us to construct a cohesive picture of the temperature dependence of *T*_1_ and suggests metal–donor covalency and nuclear spin content as key design principles for elongating spin–lattice relaxation in qubit candidates. At the lowest temperatures, the vanadium complexes, **1′** and **3′**, display a longer *T*_1_ than their copper analogues, **2′** and **4′**. This observation is supported by the decreased susceptibility of the vanadium complexes to the direct process as determined by the variable-field ac susceptibility data and is a consequence of their weaker electron–nuclear hyperfine coupling. Above 10 K the copper complexes display longer *T*_1_ values than their vanadium counterparts, and the dithiolates possess longer *T*_1_ values than their selenium analogues. To understand the higher temperature dynamics, we must consider both the effective spin–orbit coupling experienced by the unpaired spin as well as the local modes of vibration.

Ligand-based spin–orbit coupling may become significant in governing *T*_1_ in highly covalent systems. In the selenium–donor complex **4**, spin delocalization away from the metal could significantly weaken the effective spin–orbit coupling experienced by the electronic spin.^51^,^52^ This in turn suppresses the Raman process and yields longer *T*_1_'s.^53^ However, **4** displays a faster spin–lattice relaxation rate than its sulfur analogue, **2**. This unexpected hastening of relaxation time may be the result of the increased spin–orbit coupling from the selenium–donor atoms.^54^,^55^ This prompts us to conclude the heavy atom effect from donor atoms strongly affects *T*_1_ in highly covalent systems. Finally, the combined spin–orbit coupling of the metal-based spin with the low-energy vibrational modes in the vanadium(iv) complexes accounts for their fast *T*_1_ rates at higher temperatures. In contrast, reduction of spin–orbit coupling induced by metal–ligand covalency in the copper complexes, specifically **2**, in combination with their higher energy local vibrational modes fortifies *T*_1_ up to room temperature.

## Outlook

The systematic study reported herein provides the first direct evidence of a dependence of *T*_1_ on metal–ligand covalency. Specifically, longer *T*_1_ relaxation times for the sulfur donors in **1** and **2** in relation to their selenium analogues, **3** and **4**. Importantly, such covalency appears to engender a significant role for spin-delocalization in copper complexes **2** and **4**, enabling *T*_1_, and consequently, *T*_2_ to persist until room temperature. The significant role of M–L covalency ultimately dictates the potential for the observation of spin coherence at higher temperatures and must be carefully considered when designing molecular qubit candidates. Designing qubit candidates that persist to room temperature offers potential for creating designer qubits for quantum sensing applications within biology. Beyond quantum information science, these results may see impact in dynamic nuclear polarization where spin–lattice relaxation is an important parameter.^56^

## Conflicts of interest

There are no conflicts to declare.

## Supplementary Material

Supplementary information

Crystal structure data
