# Supplementary material for: Metal–ligand covalency enables room temperature molecular qubit candidates
Source: Chem Sci. 2019 May 31;10(27):6707–14. doi: 10.1039/c9sc00074g (PMC6625489; doi:10.1039/c9sc00074g)
Supplement: Supplementary file 1 [file SC-010-C9SC00074G-s001.pdf]

Electronic Supplementary Material (ESI) for Chemical Science.
This journal is © The Royal Society of Chemistry 2019

Electronic Supplementary Information for:

Metal-Ligand Covalency Enables Room Temperature Molecular Qubit Candidates

Majed S. Fataftah,[1] Matthew D. Krzyaniak,[1,2] Bess Vlaisavljevich,[3] Michael R. Wasielewski,[1,2*] Joseph M. Zadrozny,[4*] and Danna E. Freedman[1*]

[1] *Department of Chemistry, Northwestern University, Evanston, Illinois 60208.*

[2] *Institute for Sustainability and Energy at Northwestern, Northwestern University, Evanston, Illinois 60208.*

[3] *Department of Chemistry, University of South Dakota, Vermillion, South Dakota 57069.*

[4] *Department of Chemistry, Colorado State University, Fort Collins, Colorado, 80523.*

Table of Contents

**Full Experimental Details**	**S4**
**Table S1** | Summary of crystallographic data for **1**	**S15**
**Table S2** | Summary of crystallographic data for **3**	**S16**
**Table S3** | Summary of crystallographic data for **4**	**S17**
**Table S4** | Summary of crystallographic data for **5**	**S18**
**Table S5** | Summary of crystallographic data for **6**	**S19**
**Table S6** | Summary of crystallographic data for **7**	**S20**
**Table S7** | Summary of crystallographic data for **8**	**S21**
**Table S8** | Select structural parameters for **1–4**	**S22**
**Table S9-S10** | Variable-temperature Cole-Cole fitting parameters for **1** and **2**	**S22**
**Table S11-S12** | Variable-temperature Cole-Cole fitting parameters for **3** and **4**	**S23**
**Table S13-S14** | Variable-field Cole-Cole fitting parameters for **1** and **2**	**S24**
**Table S15-S16** | Variable-field Cole-Cole fitting parameters for **3** and **4**	**S25**
**Table S17-S18** | Saturation recovery fitting parameters for **1′** and **2′**	**S26**
**Table S19-S20** | Saturation recovery fitting parameters for **3′** and **4′**	**S27**
**Table S21** | T_2 recovery fit parameters for **1′–4′**	**S28**
**Table S22** | Variable field ac susceptibility fit parameters for **1–4**	**S29**
**Table S23** | M-E bond distances for the DFT optimized geometries	**S30**
**Table S24** | NBO spin densities at the metal center	**S30**
**Table S25** | MS-CASPT2 ***g***-tensors for structures **1–4**	**S30**
**Table S26** | Tabulated intramolecular vibrational modes of **1–4**	**S31**
**Figure S1** | Thermal ellipsoid depictions of **1–4**	**S32**
**Figure S2** | Infrared spectroscopy data for **1–4**	**S33**
**Figure S3** | Electronic absorption spectra for **1–4**	**S34**
**Figure S4** | Electrochemical data for **1–4**	**S35**
**Figure S5** | Cw EPR spectra of **1′–4′**	**S36**
**Figure S6** | Variable temperature ac magnetic susceptibility data of **1**	**S37**
**Figure S7** | Variable temperature ac magnetic susceptibility data of **2**	**S38**
**Figure S8** | Variable temperature ac magnetic susceptibility data of **3**	**S39**
**Figure S9** | Variable temperature ac magnetic susceptibility data of **4**	**S40**
**Figure S10** | Variable field ac magnetic susceptibility data of **1**	**S41**
**Figure S11** | Variable field ac magnetic susceptibility data of **2**	**S42**

Figure S12 | Variable field ac magnetic susceptibility data of **3** **S43**
Figure S13 | Variable field ac magnetic susceptibility data of **4** **S44**
Figure S14 | Variable temperature X-band pulsed EPR (T_1 & T_2) data of **1′** **S45**
Figure S15 | Variable temperature X-band pulsed EPR (T_1 & T_2) data of **2′** **S46**
Figure S16 | Variable temperature X-band pulsed EPR (T_1 & T_2) data of **3′** **S47**
Figure S17 | Variable temperature X-band pulsed EPR (T_1 & T_2) data of **4′** **S48**
Figure S18 | Overlay of T_1 and τ for **1–4** **S49**
Figure S19 | T_1 comparative fits of spectral diffusion and stretched exponential (**1′**&**3′**) **S50**
Figure S20 | T_1 comparative fits of spectral diffusion and stretched exponential (**2′**&**4′**) **S51**
Figure S21 | Fits to the temperature dependent T_1 in **1′–4′** **S52**
Figure S22 | Plots of spin densities from M06-L calculation for **1–4** **S53**
Figure S23 | State-averaged active orbitals from MS-CASPT2 calculations for **1** and **3** **S54**
Figure S24 | State-averaged active orbitals from MS-CASPT2 calculations for **2** and **4** **S55**
Figure S25 | Overlay of the computational calculated vibrational modes for **1–4** **S56**
Figure S25 | Computed van der Waals phonon density of states for **2–4** **S57**
References **S58**

Full Experimental Details

General considerations. All manipulations and syntheses were performed under a N_2 atmosphere with either an MBraun Unilab Pro glovebox, Vacuum Atmosphere Nexus II glovebox, or Schlenk techniques. Glassware was either oven-dried at 150 °C for at least four hours and/or flame-dried prior to use. Acetonitrile (MeCN), methanol (MeOH), and diethylether (Et_2O), and dimethylformamide (DMF) were dried using a commercial solvent purification system from Pure Process Technology and stored over 3 or 4 Å sieves prior to use. Et_2O was subjected to a test with a standard purple solution of sodium benzophenone ketyl in THF to confirm low O_2 and H_2O content prior to use. Triethylamine (Et_3N), pyridine, and catechol were purified prior to use according to the literature procedures.[1] 1,2-benzenedithiol,[2] 1,2-benzenediselenol,[3] vanadyl acetoacetonate ($VO(acac)_2$)[4], and tetrakis-dimethylamide titanium(IV)[5] were prepared following the literature procedures. All other reagents were used as received.

$(Ph_4P)_2[V(C_6H_4S_2)_3]$ (1). A 50 mL Schlenk flask was charged with a magnetic stirbar, 1,2-benzenedithiol (350 mg, 2.46 mmol), $VO(acac)_2$ (215 mg, 0.81 mmol), $(Ph_4P)Br$ (664 mg, 1.58 mmol), 10 mL of MeCN and 0.25 mL of Et_3N. We heated the resulting dark red/purple mixture at reflux for 8 hours then allowed it to cool to room temperature. Filtration of the reaction mixture afforded a dark red solid (**1**) that was washed with 10 mL of Et_2O and dried *in vacuo* (831 mg, 91 %). Crystals suitable for single-crystal X-ray diffraction were obtained by diffusion of Et_2O vapor into a solution of **1** in MeCN. IR (cm^{-1}): 3051, 1583, 1537, 1480, 1430, 1415, 1315, 1280, 1228, 1187, 1151, 1101, 1020, 995, 928, 855, 755, 717, 686, 664, 616, 522, 480, 454, 432, 394, and 366; see Figure S2 for spectrum. UV-vis (MeCN); $\tilde{\nu}_{max}$, cm^{-1} (ε_M, $M^{-1}cm^{-1}$): 11376 (4530), 18020 (7900), 23752 (9344), and 30211 (17095); see Figure S3 for spectrum. ESI/MS (*m*/*z*): $\{V(C_6H_4S_2)_3\}^{1-}$, 470.821 (base), and $\{(Ph_4P)[V(C_6H_4S_2)_3]\}^{-}$, 809.851. Combustion analyses calculated for $C_{66}H_{52}P_2S_6V$ (found): 68.91 (69.19) %C; 4.56 (4.59) %H.

$(Ph_4P)_2[Cu(C_6H_4S_2)_2]$ (2). We employed a modified literature procedure following a similar route to a previous report.[6] In the glovebox, a magnetic stirbar, $CuCl_2$ (65 mg, 0.48 mmol) and 1,2-dithiobenzene (145 mg, 1.02 mmol) were combined in 15 mL of DMF at room temperature in a 20 mL scintillation vial that resulted in a light green solution. The addition of 2 mL of Bu_4NOH (1 M in THF, 2.0 mmol) induced an immediate a color change to dark red. The solution mixture was stirred for 30 minutes prior to the addition of $(Ph_4P)Br$ (420 mg, 1.00 mmol). The reaction mixture was stirred for an additional 2 hours prior to being stored overnight at −35 °C. Filtration of the reaction mixture afforded a dark red, microcrystalline solid (**2**) that was washed with 10 mL of Et_2O and dried *in vacuo* (440 mg, 91 %). Crystals suitable for single-crystal X-ray diffraction were obtained by layering Et_2O on top of a solution of **2** in DMF. IR (cm^{-1}): 3034, 2981, 1582, 1555, 1537, 1480, 1433, 1411, 1368, 1335, 1317, 1268, 1231, 1201, 1186, 1158, 1097, 1046, 1021,

993, 948, 919, 850, 758, 736, 717, 783, 658, 615, 519, 468, 450, and 438; see Figure S2 for spectrum. UV-vis (DMF); $\tilde{v}_{max}$ (ε_M, $M^{-1}cm^{-1}$): 16806 (392), 21690 (5000), 26525 (7130), 29325 (9650); see Figure S3 for spectrum. ESI/MS (*m/z*): $\{H[Cu(C_6H_4S_2)_2]\}^-$, 342.921 (base); $\{(Ph_4P)_2[Cu(C_6H_4S_2)_2]\}^-$, 1024.206. Combustion analyses calculated for $C_{60}H_{48}S_4P_2Cu$ (found): 70.46 (70.06) %C; 4.73 (4.78) %H.

$(Ph_4P)_2[V(C_6H_4Se_2)_3]$ (3). A 25 mL Schlenk flask was charged with $VO(acac)_2$ (80 mg, 0.33 mmol), $(Ph_4P)Br$ (275 mg, 0.66 mmol), and 5 mL MeCN. A solution of 1,2-benzenediselenol (230 mg, 0.99 mmol) in 3 mL MeCN was added, immediately producing a dark colored solution. Triethylamine was added (0.25 mL) and the resulting mixture was refluxed overnight. The mixture was allowed to cool, then filtered, affording **3** as a dark solid (387mg, 86 %). Single crystals suitable for single-crystal X-ray diffraction were obtained by diffusion of Et_2O vapor into a solution of **3** in MeCN. IR (cm^{-1}): 3051, 1583, 1537, 1480, 1430, 1415, 1315, 1280, 1228, 1187, 1151, 1101, 1020, 995, 928, 855, 755, 717, 686, 664, 616, 522, 480, 454, 432, 394, and 366; see Figure S2 for spectrum. UV-vis (MeCN); $\tilde{v}_{max}$ (ε_M, $M^{-1}cm^{-1}$): 10142 (3285), 16400 (5409), 22522 (7631), 29586 (11717) and 37037 (38696); see Figure S3 for spectrum. ESI/MS (*m/z*): $\{V(C_6H_4Se_2)_3\}^{1-}$, 754.47 (base), $\{(Ph_4P)[V(C_6H_4Se_2)_3]\}^{1-}$, 1093.46, and $\{V(C_6H_4Se_2)_3\}^{2-}$, 377.305. Combustion analyses calculated for $C_{66}H_{52}P_2Se_6V$ (found): 55.37 (55.13) %C; 3.66 (3.47) %H.

$(Ph_4P)_2[Cu(C_6H_4Se_2)_2]$ (4). In a scintillation vial in the glovebox, a magnetic stirbar, $CuCl_2$ (65 mg, 0.48 mmol) and 1,2-diselenobenzene (244 mg, 1.03 mmol) were combined with 15 mL of DMF at room temperature giving rise to a light green solution. Following the addition of 2 mL of Bu_4NOH (1 M in THF, 1 mmol), the solution rapidly changed color to dark red. The solution mixture was further stirred for 30 minutes prior to the addition of $(Ph_4P)Br$ (420 mg, 1.0 mmol). The reaction mixture was stirred for an additional 4 hours prior to being stored overnight in at −35 °C. Filtration of the reaction mixture afforded a very dark red, microcrystalline solid (**4**) that was washed with 5 mL of cold Et_2O and dried *in vacuo* (322 mg, 53.2 %). Crystals suitable for single-crystal X-ray diffraction were obtained by layering Et_2O on top of a solution of **4** in DMF. IR (cm^{-1}): 3036, 2983, 1582, 1551, 1537, 1480, 1433, 1409, 1384, 1335, 1315, 1266, 1230, 1201, 1187, 1160, 1103, 1080, 1025, 993, 975, 840, 752, 738, 717, 680, 640, 615, 519, 466, 450, 432, and 411; see Figure S2 for spectrum. UV-vis (MeCN); $\tilde{v}_{max}$ (ε_M, $M^{-1}cm^{-1}$): 15600 (910), 20490 (6995), 25500 (3800), 29070 (7300); see Figure S3 for spectrum. ESI/MS (*m/z*): $\{H[Cu(C_6H_4Se_2)_3]\}^-$, 534.675 (base). Combustion analyses calculated for $C_{60}H_{48}Se_4P_2Cu \cdot DMF_{0.2}$ (found): 58.82 (58.65) %C; 3.95 (4.10) %H.

$(Ph_4P)_2[Ti(C_6H_4S_2)_3]$ (5). In the glovebox, a magnetic stirbar, $Ti(NMe_2)_4$ (52 mg, 0.26 mmol) and 1,2-dithiobenzene (95 mg, 0.67 mmol) were combined in 15 mL of THF at −78 °C triggering an immediate color change to dark red. The solution mixture was stirred for 2 hours, followed by the addition of $(Ph_4P)Br$ (190 mg, 0.45 mmol). The reaction mixture was stirred for an additional 1 hour prior to being stored overnight in at −35 °C. Filtration of the reaction mixture afforded a very dark red, microcrystalline solid (**5**) that was washed with 5 mL of cold Et_2O and dried *in vacuo* (212 mg, 74 %). Crystals suitable for single-crystal X-ray diffraction were obtained by layering Et_2O on top of a solution of **5** in DMF. IR (cm^{-1}): 3052, 2958, 2771, 2426, 1582, 1560, 15341, 1480, 1462, 1431, 1337, 1315, 1272, 1231, 1185, 1156, 1160, 1100, 1046, 1021, 997, 928, 885, 856, 811, 756, 720, 687, 660, 615, 524, 481, 454, 434, and 414. UV-vis (MeCN); $\tilde{\nu}_{max}$ (ε_M, $M^{-1}cm^{-1}$): 17500 (7000), 23000 (14000), 28950 (10500). ESI/MS (*m*/*z*): $\{[Ti(C_6H_4S_2)_3]\}^{2-}$, 243.266 (base); $\{(Ph_4P)[V(C_6H_4S_2)_3]\}^{-}$, 807.523. Combustion analyses calculated for $C_{66}H_{52}S_6P_2Ti$ (found): 69.09 (69.32) %C; 4.57 (4.54) %H.

$(Ph_4P)_2[Ni(C_6H_4S_2)_2]$ (6). In a 20 mL scintillation vial in the glovebox, a magnetic stirbar, $NiCl_2$ (65 mg, 0.5 mmol) and 1,2-dithiobenzene (150 mg, 1.06 mmol) were combined in 15 mL of DMF at room temperature to yield a green solution. The addition of 2 mL of Bu_4NOH (1 M in THF, 1.0 mmol) induced a color change to dark red. The solution mixture was stirred for an additional 20 minutes prior to the addition of $(Ph_4P)Br$ (419 mg, 1.00 mmol). The reaction mixture was stirred for an additional 4 hours prior to being stored overnight in at −35 °C. Filtration of the reaction mixture afforded a dark reddish purple, microcrystalline solid (**6**) that was washed with 5 mL of Et_2O and dried *in vacuo* (380 mg, 75 %). Crystals suitable for single-crystal X-ray diffraction were obtained by layering Et_2O on top of a solution of **6** in DMF. IR (cm^{-1}): 3034, 2981, 1582, 1550, 1540, 1480, 1431, 1413, 1335, 1317, 1282, 1229, 1187, 1158, 1101, 1046, 1017, 993, 946, 917, 842, 758, 734, 717, 683, 664, 615, 520, 468, 450 and 434; UV-vis (DMF); $\tilde{\nu}_{max}$ (ε_M, $M^{-1}cm^{-1}$): 10950 (1820), 24270 (10000), 31800 (36000); see Figure S3 for spectrum. ESI/MS (*m*/*z*): $\{[Ni(C_6H_4S_2)_3]\}^{-}$, 337.925 (base); $\{(Ph_4P)_2[Ni(C_6H_4S_2)_3]\}^{-}$, 1017.101. Combustion analyses calculated for $C_{60}H_{48}Se_4P_2Cu$ (found): 70.80 (70.64) %C; 4.75 (4.93) %H.

$(Ph_4P)_2[Ti(C_6H_4Se_2)_3]$ (7). Similar to **5**, a magnetic stirbar, $Ti(NMe_2)_4$ (54 mg, 0.27 mmol) and 1,2-diselenobenzene (162 mg, 0.69 mmol) were combined in 15 mL of THF at −78 °C in a nitrogen glovebox, triggering an immediate solution color change to a very dark green. The solution mixture was stirred for 2 hours, followed by the addition of $(Ph_4P)Br$ (209 mg, 0.5 mmol). The reaction mixture was stirred for an additional 1 hour prior to being stored overnight in at −35 °C. Filtration of the reaction mixture afforded a very dark green, microcrystalline solid (**7**) that was washed with 5 mL of Et_2O and dried *in vacuo* (313 mg, 87 %). Crystals suitable for single-crystal X-ray diffraction were obtained by layering Et_2O on top of a

solution of **7** in DMF. IR (cm^{-1}): 3050, 3020, 2981, 1582, 1551, 1541, 1480, 1425, 1413, 1337, 1313, 1266, 1229, 1186, 1158, 1103, 1080, 1027, 995, 931, 856, 754, 717, 685, 640, 615, 522, 454, and 433. UV-vis (DMF); $\tilde{\nu}_{max}$ (ε_M, $M^{-1}cm^{-1}$): 10845 (1824), 16025 (6810), 21050 (13570), 27800 (11700), 33240 (19700). ESI/MS (*m*/*z*): $\{[Ti(C_6H_4Se_2)_3]\}^{2-}$, 376.630 (base); $\{(Ph_4P)[Ti(C_6H_4Se_2)_3]\}^{-}$, 1080.766. Combustion analyses calculated for $C_{66}H_{52}S_6P_2Ti$ (found): 55.49 (55.39) %C; 3.67 (3.76) %H.

$(Ph_4P)_2[Ni(C_6H_4Se_2)_2]$ (8). In the glovebox, a 20 mL scintillation vial was charged with a magnetic stirbar, $NiCl_2$ (63 mg, 0.48 mmol) and 1,2-diselenobenzene (240 mg, 1.02 mmol) in 15 mL of DMF at room temperature to yield a green solution. 2 mL of Bu_4NOH (1 M in THF, 1.0 mmol) was added and induced the solution to change color to very dark red. The solution mixture was stirred for 30 minutes prior to the addition of $(Ph_4P)Br$ (422 mg, 1.01 mmol). The reaction mixture was stirred for an additional 4 hours prior to being stored overnight in at −35 °C. Filtration of the reaction mixture afforded a very dark red, almost black, microcrystalline solid (**8**) that was washed with 5 mL of Et_2O and dried *in vacuo* (396 mg, 65 %). Crystals suitable for single-crystal X-ray diffraction were obtained by layering Et_2O on top of a solution of **8** in DMF. IR (cm^{-1}): 3034, 2999, 2981, 1582, 1551, 1539, 1478, 1427, 1411, 1335, 1315, 1276, 1229, 1201, 1186, 1160, 1101, 1078, 1023, 993, 952, 935, 919, 841, 752, 736, 717, 683, 644, 615, 519, 469, 452, 428 and 401; UV-vis (MeCN); $\tilde{\nu}_{max}$ (ε_M, $M^{-1}cm^{-1}$): 11510 (900), 24900 (7500), 30400 (33000); ESI/MS (*m*/*z*): $\{H[Ni(C_6H_4Se_2)_3]\}^{-}$, 527.682 (base); $\{(Ph_4P)[Ni(C_6H_4Se_2)_2]\}^{-}$, 1205.707. Combustion analyses calculated for $C_{60}H_{48}Se_4P_2Ni{\cdot}DMF_{0.3}$ (found): 58.71 (58.65) %C; 3.94 (4.15) %H.

Electron Paramagnetic Resonance Measurements. Samples were prepared for analysis by a solid-state dilution in their respective diamagnetic analogues. Solid-state dilutions were prepared in a 1:200 (0.5%) ratio to suppress the influence of intermolecular electronic spin interactions on T_2 and T_1. All samples were loaded into 4 mm OD quartz tubes (Wilmad 707-SQ-250M), restrained with eicosane, and flame-sealed under high vacuum.

All spectroscopic data were obtained with a Bruker E580 X/W-band spectrometer equipped with a split ring resonator (ER4118X-MS5) and a 1 kW TWT amplifier (Applied Systems Engineering). Prior to all measurements, the resonator was over-coupled to minimize ringdown following application of the microwave pulses. Temperature was controlled with an Oxford Instruments CF935 helium cryostat and an Oxford Instruments ITC503 temperature controller. All EPR data were processed by a combination of XEpr,[7] Matlab,[8] Easyspin,[9] and Origin.[10] Absolute intensities of the cw-EPR spectra were normalized between 0 and 1 prior to simulation using pepper in EasySpin.

Spin-lattice relaxation times were obtained using saturation recovery sequences at the highest intensity peaks in the echo-detected spectrum. These sequences achieved saturation by applying a picket-fence

saturation sequence of twenty consecutive 12 ns pulses. Following a delay time T beginning at 100 ns, Hahn-echo detection was used to monitor the recovery from saturation with $\pi/2$ and π pulses of 16 and 32 ns, respectively. Four-step phase cycling was used on these measurements. All data was phased by maximization of the sum of the square of the data in the real component of the spectrum. The data was fit to account for the influence of spectral diffusion (S), [11] using the following equation, $I = -A(e^{-\left(\frac{t}{T_1}\right) - \sqrt{\frac{t}{S}}} - I_0 - 1)$. Noteworthy, comparison of the extracted T_1 values with those using a stretched exponential decay yield very similar values, whose overlaid data is presented in Figures S19–S20.

The temperature dependence of T_1 was fit to account for the direct process, Raman process, and local modes using the following expression,

$$\frac{1}{T1} = A_{Dir}T + B_{Ram}\left(\frac{T}{\Theta_D}\right)^9 J_8\left(\frac{\Theta_D}{T}\right) + C_{Loc}\frac{e^{\Delta_{loc}/T}}{(e^{\Delta_{loc}/T} - 1)^2}$$

Where T is temperature, A_{Dir} is the coefficient for the direct process, B_{Ram} is the coefficient for the Raman process, J_8 is the transport integral,[12] θ_D is the Debye temperature, C_{loc} is the coefficient for local modes, and Δ_{loc} is the energy of the active local modes of vibration. See below for further discussion of the fitting process of the temperature dependence of T_1 across **1′–4′**.

Spin-spin relaxation times (T_2) were obtained using a two-pulse Hahn echo sequence, $\pi/2$-τ-π-τ-*echo*, where τ is the time delay between pulses, and $\pi/2$ or π denote microwave pulses, 16 ns and 32 ns, respectively. Starting delay times were selected to minimize the effects of any observed ringdown. Generally, these delays were within the range of 100 to 160 ns for all complexes. Four step phase cycling was employed for these experiments as well. The decay curves were modelled with a monoexponential decay, $I = Ae^{\left(\frac{2\tau - \tau_0}{T_2}\right)} - I_0$. Fits accounting for the damping oscillation arising from ESEEM give rise to nearly identical T_2 times extracted from their mono-exponential fits (within ~5% error).

Magnetic Susceptibility Measurements. Direct-current (dc) and alternating-current (ac) susceptibility measurements were performed with a Quantum Design MPMS-XL SQUID magnetometer. Samples of **1**–**4** were prepared under an inert atmosphere as finely ground microcrystalline powders in either polycarbonate gelcaps or sealed in quartz tubes. All samples were restrained with eicosane to ensure good thermal contact with the thermal bath. Dc susceptibility measurements were performed at fields of 1000 Oe to corroborate the $S = 1/2$ spin state determined by EPR. Verification of the $S = 1/2$ spin state was determined by leveling off of the value of $\chi_M T$ at 0.370, 0.372, 0.374, and 0.377 cm^3K/mol for **1**–**4**, respectively, which

are close to the expected value of 0.375 cm^3K/mol for an $S = {}^1/_2$ ion. Low temperature data were corrected for the diamagnetic contributions of the sample holder and the sample itself through the use of Pascal's constants.[13] Magnetization curves (*M* vs. *H*) were recorded at 100 K, from 0–4 T to check for the presence of unwanted ferromagnetic impurities. Ac magnetic susceptibility experiments utilized an oscillating field of 4 Oe amplitude and frequencies from 0.1 to 1500 Hz in the field range of 0.025 – 3.5 T, and in the temperature range of 5–30 K, above which the samples relaxed at rates beyond the limits of the SQUID magnetometer (1500 Hz, or ~100 μs).

The variable-field ac magnetic susceptibility was modeled using the Brons-van Vleck equation,[14]

$$\tau^{-1} = cB^4 + d\frac{1 + eB^2}{1 + fB^2},$$

where τ is the relaxation time extracted by the generalized Debye model,[15,16] B is the applied external magnetic field, c is the coefficient to the direct process, d the zero-field relaxation rate, e relates to the spin-concentration of the sample, and f represents the ability of the external magnetic field to suppress spin-spin cross relaxation. Interestingly, e correlates with the unit cell concentration throughout the series. Compounds **2** and **4** crystallize in the same space group, $P2_1/n$, and fitting the variable-field ac data yields identical values for e. Similarly, in compounds **1** and **3**, which crystallize in P-1 and C2/c respectively, yield values of e roughly a factor of 2 larger in **3** versus **1** and may relate to the density of spin-centers in the unit cell.

X-ray Structure Determination. All diffraction data were collected in the X-ray crystallography lab of the Integrated Molecular Structure Education and Research Center (IMSERC) at Northwestern University. Single crystals of **1**–**8** suitable for X-ray diffraction analysis were coated in Paratone N oil and mounted on a MiTeGen MicroLoop™. Crystallographic data for **4**, **5**, and **8** were collected on a Bruker KAPPA diffractometer equipped with a MoKα sealed tube diffraction source with a graphite monochromator, and a Bruker APEX II detector. Data for all other compounds (**1**, **3**, **6**, and **7**) were acquired on a Bruker KAPPA diffractometer equipped with a MoKα IμS microfocus X-ray source with Quazar Optics and a Bruker APEX II detector. All datasets were collected at 100 K, except for **4** collected at 250 K, and temperature was controlled via an Oxford Cryosystems Cryostream. Raw data were integrated and corrected for Lorentz and polarization effects with SAINT v8.27B.10[17] Absorption corrections were applied using SADABS.[18] Space group assignments were determined by examination of systematic absences, E-statistics, and successive refinement of the structures. Structures were solved using direct methods in SHELXT and further refined with SHELXL-2013[19] operated with the OLEX2 interface.[20] Hydrogen atoms were placed in ideal positions and refined using a riding model for all structures.

Other Physical Measurements. Combustion analyses were performed by Midwest Microlab Inc. (Indianapolis, IN). Infrared spectra were recorded on a Bruker Alpha FTIR spectrometer equipped with an attenuated total reflectance accessory and diamond anvil. Electrospray ionization mass spectrometry measurements were performed on MeCN solutions of **1**–**8** with Bruker AmaZon SL ESI-Ion Trap Mass Spectrometers at the IMSERC facility of Northwestern. UV-Vis spectra were collected on MeCN or DMF solutions of **1**–**8** with a Varian Cary 5000 spectrophotometer. Electrochemical data in the form of cyclic voltammograms are depicted in Figure S4 and were collected under an inert atmosphere with a CHI 760c potentiostat. Measurements were performed using a standard one-compartment cell, a glassy carbon working electrode, platinum wire counter electrode, and silver wire reference electrode. Typical samples for electrochemical analysis utilized 0.1 mM solutions of $(Bu_4N)PF_6$ supporting electrolyte and a $[Cp_2Fe]^{0/1+}$ internal reference.

Determination of Covalency Parameter, α^2. The covalency parameter was extracted according to the two equations below following the method by McGarvey,[21,22]

$$A_{\parallel} = P(-\kappa - \frac{4}{7}\alpha^2 + \Delta g_{\parallel} + \frac{3}{14}\Delta g_{\perp})$$

$$A_{\perp} = P(-\kappa + \frac{2}{7}\alpha^2 + \frac{11}{14}\Delta g_{\perp})$$

Using A and g parameters determined by simulating the cw-EPR spectra of **2** and **4**, α^2 values of 0.51 and 0.395 were extracted for **2** and **4**, respectively. The parameters were calculated using a P value of 0.0388 cm^{-1} as previously determined for square planar Cu^{2+} complexes.[23]

Additional information on fitting variable temperature T_1. The relaxation mechanisms we believe to be operative in compounds **1′**–**4′** are the direct process, the Raman process, and local modes of vibration. Given all compounds are S = ½, it is unrealistic to assign an Orbach mechanism, which necessitates accessible low-lying spin states only available in higher spin systems or coupled systems.[24] It is important to note the significant errors associated with the fit parameters extracted from fitting the temperature dependence of T_1. This is the result of utilizing models that were derived for higher symmetry solids, such as the Debye model, and poorly represent the complex phonon structure present in molecular solids. Furthermore, the parameters extracted for local modes during fitting the temperature dependence of T_1 represents some weighted average of all local modes contributing to spin-lattice relaxation, and the inclusion of further terms leads to problematic overparameterization.[25] Furthermore, the strong dependence of the fits on all parameters in the expression for the relaxation rate leads to rather large error bars during

the fitting process. However, comparison of fits across the series of **1–4** allows us to corroborate our qualitative analysis of the T_1 relaxation mechanisms and deduce important chemical insight.

Close inspection of the parameters across the series reveals a number of interesting comparisons. The Raman and Local mode coefficients, B_{Ram} and C_{Loc}, depend strongly on the ability of the spin to couple to the phonon bath and vibrational modes. This coupling is strongly dictated by spin-orbit coupling (SOC). Interestingly, B_{Ram} is relatively invariant between **1** and **3** (~5×10^5), whereas it changes by an order of magnitude between **2** and **4** (1.4×10^5 vs 2×10^6). The more dramatic change in B_{Ram} between the vanadium and copper complexes arises from the influence of M-L covalency influencing the effective SOC experienced by electronic spin; in **4**, the electronic spin experiences the combined SOC of the Cu^{2+} and Se heavy atoms more dramatically relative to **3** because the electronic spin primarily resides on V^{4+} centre in **3**. Compound **2** experiences the weakest SOC across the series owing the orbital reduction factor arising from delocalization of the spin onto the lighter S-donor atoms. This fact is mirrored in the smallest values of B_{Ram} and C_{Loc} across the series.

Structural Characterization. Comparison of the average M–L bond distances in **1–4** reveal that the metal-donor bond distances are all well below those computed using the Shannon-Prewitt ionic radii for Cu^{2+}, V^{4+}, S^{2-}, Se^{2-}, with larger deviations observed for the Cu^{2+} complexes (see Table S8).[26] This suggests enhanced metal-donor covalency in the copper complexes relative to their vanadium counterparts.

Additional ac susceptibility analysis. Alternating current (ac) magnetic susceptibility measurements reveal a peak in the out-of-phase response at a given oscillating field frequency, static magnetic field strength, and temperature, and yields the rate of spin-lattice relaxation, $1/\tau$. Variable temperature (from 5 to 25 K) ac experiments were performed on bulk, microcrystalline samples of **1–4** at 3400 G to best compare with our pulsed EPR T_1 data (Figures S6–S9). It is worth noting that the temperature range of inspection by ac susceptibility is limited to relaxation times that span between 1.6 s to 100 μs owing to instrumental constraints. Thus, we only access a select portion of the relaxation window observed by our pulsed EPR measurements. Regardless, our variable temperature ac susceptibility measurements closely reproduce the relaxation times extracted by EPR. Comparison of the temperature dependence of τ with T_1 reveals discrepancies, with the largest divergence apparent in **3**. These data reproduce an important result from the pulsed data: longer T_1 parameters for **2** and **4** relative to their vanadium analogues **1** and **3**. What they don't reproduce, however, is a longer T_1 for the diselenocatecholate complexes **3** and **4** at 5 K. We hypothesize these differences originate from cross-relaxation, as each measurement is performed at a different

extreme of spin concentration. Here, electronic spin-spin dipolar coupling provides an additional mechanism of spin-lattice relaxation that gives rise to the discrepancy between T_1 and τ across the temperature range of inspection. Further, the discrepancy between T_1 and τ in our measurements is exacerbated by the application of a saturation recovery pulse sequence to measure T_1. This suppresses spectral diffusion, whose physical mechanism involves cross-relaxation between adjacent dipolar coupled spins. We believe cross-relaxation is the likely culprit for explaining the discrepancy between τ and T_1 across temperature, exacerbated by the fact the temperature dependence of spectral-diffusion remains poorly understood.

The variable-field ac magnetic susceptibility measurements were performed to reveal additional mechanistic information regarding cross-relaxation. Specifically, the variable-field ac measurements permit deconvolution of the direct process and spin-spin cross relaxation that are often dominant at the lowest temperatures of measurement. The variable-field ac data for **1**–**4** reveals drastically different relaxation times. However, modeling the data according to the Brons van-Vleck model, a phenomenological model developed to describe the field-dependence of relaxation for S = ½ systems reveals important mechanistic information. In this model, the parameter c relates to the susceptibility of the spin to the direct process, and trends proportionally with the isotropic hyperfine interactions. This is expected given that the direct process is mediated by mixing of the M_S = ±½ states, in this case by hyperfine interactions. Further, both the e and f parameters are dictated by a variety of spin-spin interactions, such as dipolar coupling, exchange interactions, and hyperfine interactions, and should not be discussed independently from one another. While f represents the square of the internal magnetic field within the sample and e relates to the spin concentration in the sample, a direct comparison across the series may be more adequately seen by considering the ratio f/e across the series, thereby normalizing f with regards to e (14, 42.3, 3.1, and 17.7 for **1**–**4** respectively). Here, a more dramatic reduction in f/e takes place between **1** and **3** relative to **2** and **4**, by factors of 4.5 and 2.38, respectively. We attribute this discrepancy to the dipolar interactions, whereby weaker dipolar coupling to the ^{77}Se nuclei in **3** relative to **4** result in a more dramatic reduction in f/e for **3** relative to **4**. This in turn further hinders the ability of the magnetic field to suppress cross-relaxation. This discrepancy is primarily attributable to the enhanced M-L covalency in **4** relative to **3**.

Additional T_2 analysis. Returning to our analysis of the trends in T_2 requires addressing both M–L covalency and the nuclear spins located on the ligands. Prior work from our group and others indicates that ligand-based spins located outside a 6 Å radius from vanadium(IV) ions limit T_2.[27,28] Within this radius, the spin-diffusion barrier radius, the nuclear spins are strongly dipolar coupled

to the electronic spin and do not participate in flip-flops that shorten T_2. These studies also highlight that such nuclear spins become more effective at limiting T_2 with increasing distance past 6 Å. Complexes **1′–4′** all possess ^{1}H nuclear spins at relatively fixed distances from the metal ions, and importantly, these distances are all over 6 Å, indicating the protons may be suppressing T_2. However, the time scale of T_2 at the ~20 K peak goes against the expected trend, in that T_2 here increases as the protons move further away in complexes **1′–3′**. Furthermore, the longest T_2 is held by **4′**, while it neither possesses the longest nor shortest M···H distances out of all the compounds measured. The unexpected trend observed in **1′–3′** likely indicates the importance of factors other than merely distance playing a role within the diffusion barrier radius. One such factor is spin delocalization away from the metal ion – M-L covalency – and this may explain the behavior of **1′–3′**, as well as the outlying behavior of **4′**.

The relationship between covalency and the diffusion barrier radius is not established, though it can be readily envisioned: diffusion of spin density onto the ligands in **3** and **4** could engender a stronger dipolar coupling with ligand-based protons, effectively bringing them within the spin-diffusion radius around the metal center. This would in turn suppress nuclear spin flip-flops and prolong low-temperature T_2's, while simultaneously making the electronic spin more sensitive to adjacent molecular spins and counterion nuclear spins. Evidence of diffusion of spin density onto the catecholate ligands is corroborated by ^{77}Se hyperfine coupling in **4** and our theoretical calculations, and previous calculations which support significant thiyl character in **3**. The origin of the increase in T_2 between 5 and 20 K is an anomaly, and an exact explanation of this behavior is a presently unanswered question.

Computational Details

State averaged complete active space self-consistent field (SA-CASSCF) and multistate complete active space second-order perturbation theory (MS-CASPT2) calculations were performed for the first five excited states of **1**, **2**, **3**, and **4**. Calculations were performed on the geometries obtained by diffraction experiments. Structures **1** and **3** are V^{IV} complexes and are $3d^1$; therefore, the active space was chosen to include the five 3d orbitals and the single electron, (1*e*, 5*o*). The Cu^{II} complexes (**2** and **4**) are $3d^9$. It is well-established that when performing CASPT2 calculations on first row transition metals with a more than half full *d*-shell, the 4d orbitals must also be included in the active space. Therefore, for these complexes the minimal active space was taken to be (9*e*,10*o*). However, complexes 3 and 4 have a covalent interaction between the d_{x2-y2} orbital and the S (or Se) 2p orbitals. For our best active space, this orbital was also included leading to an active space of (11*e*,11*o*). No symmetry was imposed in the calculations. The second-order Douglas-Kroll-

Hess (DKH) Hamiltonian was used in all calculations along with ANO-RCC basis sets. Basis sets of triple-ζ quality were used for Cu, V, S, and Se, a double-ζ basis was used for C, and a minimal basis set was used for H. Specifically, the following contractions were employed: Cu: 6s5p3d2f1g, V: 6s5p3d2f1g, S: 5s4p2d1s, Se: 6s5p3d1f, C: 3s2p1d, and H: 1s. Cholesky decomposition and local exchange screening were used in the computation of the integrals. All calculations were performed in Molcas 8.0.

The EPR *g*-tensor was calculated. First, the restricted active space state interaction (RASSI) approach was applied to include coupling between the five CASPT2 spin free states. MS-CASPT2 energies were used as the diagonal elements in the effective Hamiltonian. Subsequently the SINGLE_ANISO module was employed to compute the magnetic properties. Additionally, geometry optimizations were performed using density functional theory (DFT). Specifically, the M06-L and B3LYP functionals were used to optimized structures **1**–**4** using the Gaussian program package. The def2-TZVP basis set was used for all atoms. The SMD solvation model was used to model the DMF solvent. Harmonic vibrational frequencies were computed both to confirm the structures as minima and to analyze the bond stretching and bending motions between the M-E groups. Natural bond orbital analysis and spin densities was computed using the M06-L functional.

Phonon density of states calculations for **2**–**4** were performed using the phonopy code to generate the phonon modes from displacements on the DFT optimized structures. I optimized the geometry using the VASP software package and the rev-vdW-DF2 functional. I used an energy cut off-of 550 eV in the plane wave basis set. I converged the energy to 1×10^{-8} au.

Table S1 | Crystallographic information for the structural refinement of **1**.

Empirical Formula	$C_{66}H_{52}VP_2S_6$
Formula weight	1150.31 g/mol
Temperature	100(2) K
Wavelength	0.71073 Å
Crystal System	Monoclinic
Space Group	P−1
Unit Cell Dimensions	a = 13.7448(6) Å, α = 77.929(2)°
	b = 14.0207(6) Å, β = 88.111(2)°
	c = 33.4597(15) Å, γ = 60.715(2)°
Volume	5481.4(4) $Å^3$
Z	4
Density (calculated)	1.394 Mg/m^3
Absorption coefficient	0.511 mm^{-1}
F_{000}	2388.0
Crystal color	Dark Red
Crystal size	0.13 × 0.11 × 0.08 mm^3
θ range	2.36 to 26.64°
Index ranges	$-17 \leq h \leq 17$
	$-18 \leq k \leq 18$
	$-41 \leq l \leq 43$
Reflections collected	88662
Independent reflections	88500 [R_{int} = 0.0533]
Completeness to θ = 52.48°	99.8 %
Absorption correction	Multi-scan
Maximum and minimum transmission	0.655 and 0.745
Refinement method	Full-matrix least-squares on F^2
Data / restraints / parameters	25013 / 0 / 1352
Goodness-of-fit on F^{2a}	1.128
Final R indices [I > 2σ(I) = 17728 data][b]	R_1 = 5.00 %, wR_2 = 9.85 %
R indices (all data, 0.80 Å)	R_1 = 8.16 %, wR_2 = 10.90 %
Largest diff. peak and hole	0.66 and –0.35 e.$Å^{-3}$

[a] GooF = $[\Sigma[w(F_o^2–F_c^2)^2] / (n-p)]^{1/2}$ where n is the number of reflections and p is the total number of parameters refined. [b] $R_1 = \Sigma||F_o|–|F_c|| / \Sigma|F_o|$; $wR_2 = [\Sigma[w(F_o^2–F_c^2)^2] / \Sigma[w(F_o^2)^2]\ ]^{1/2}$

Table S2 | Crystallographic information for the structural refinement of **3**.

Empirical Formula	$C_{66}H_{52}VP_2Se_6$
Formula weight	1431.71 g/mol
Temperature	100(2) K
Wavelength	0.71073 Å
Crystal System	Monoclinic
Space Group	*C*2/c
Unit Cell Dimensions	a = 25.121(3) Å, α = 90.0°
	b = 13.7709(16) Å, β = 113.957(6)°
	c = 17.8697(19) Å, γ = 90.0°
Volume	5649.1(11) $Å^3$
Z	4
Density (calculated)	1.683 Mg/m^3
Absorption coefficient	0.81 mm^{-1}
F_{000}	2820.0
Crystal color	Red
Crystal size	0.11 × 0.09 × 0.08 mm^3
θ range	2.33 to 26.16°
Index ranges	$-30 \leq h \leq 29$
	$-17 \leq k \leq 14$
	$-22 \leq l \leq 22$
Reflections collected	26169
Independent reflections	25456 [R_{int} = 0.0718]
Completeness to θ = 52.48°	99.8 %
Absorption correction	Multi-scan
Maximum and minimum transmission	0.64753 and 0.7443
Refinement method	Full-matrix least-squares on F^2
Data / restraints / parameters	5663 / 0 / 235
Goodness-of-fit on F^{2a}	1.032
Final R indices [I > 2σ(I) = 4426 data][b]	R_1 = 5.66 %, wR_2 = 8.97 %
R indices (all data, 0.80 Å)	R_1 = 7.91 %, wR_2 = 15.55 %
Largest diff. peak and hole	1.01 and –0.70 e.$Å^{-3}$

[a] GooF = $[\Sigma[w(F_o^2-F_c^2)^2] / (n-p)]^{1/2}$ where n is the number of reflections and p is the total number of parameters refined. [b] $R_1 = \Sigma||F_o|-|F_c|| / \Sigma|F_o|$; $wR_2 = [\Sigma[w(F_o^2-F_c^2)^2] / \Sigma[w(F_o^2)^2]]^{1/2}$

Table S3 | Crystallographic information for the structural refinement of **4**.

Empirical Formula	$C_{60}H_{48}CuP_2Se_4$
Formula weight	1225.72 g/mol
Temperature	250(2) K
Wavelength	0.71073 Å
Crystal System	Monoclinic
Space Group	$P2_1/n$
Unit Cell Dimensions	a = 11.1972(9) Å, α = 90.0°
	b = 15.0275(12) Å, β = 94.517(2)°
	c = 15.2359(12) Å, γ = 90.0°
Volume	2555.7(4) $Å^3$
Z	2
Density (calculated)	1.593 Mg/m^3
Absorption coefficient	2.57 mm^{-1}
F_{000}	1216.0
Crystal color	Red
Crystal size	0.16 × 0.14 × 0.12 mm^3
θ range	2.57 to 29.89°
Index ranges	$-16 \leq h \leq 16$
	$-21 \leq k \leq 21$
	$-21 \leq l \leq 21$
Reflections collected	68791
Independent reflections	47934 [R_{int} = 0.0495]
Completeness to θ = 52.48°	100 %
Absorption correction	Multi-scan
Maximum and minimum transmission	0.489 and 0.746
Refinement method	Full-matrix least-squares on F^2
Data / restraints / parameters	7855 / 0 / 305
Goodness-of-fit on F^{2a}	1.037
Final R indices [I > 2σ(I) = 6612 data][b]	R_1 = 3.90 %, wR_2 = 10.92 %
R indices (all data, 0.80 Å)	R_1 = 4.92 %, wR_2 = 11.74 %
Largest diff. peak and hole	1.12 and –2.07 e.$Å^{-3}$

[a] GooF = $[\Sigma[w(F_o^2-F_c^2)^2] / (n-p)]^{1/2}$ where n is the number of reflections and p is the total number of parameters refined. [b] $R_1 = \Sigma||F_o|-|F_c|| / \Sigma|F_o|$; $wR_2 = [\Sigma[w(F_o^2-F_c^2)^2] / \Sigma[w(F_o^2)^2]]^{1/2}$

Table S4 | Crystallographic information for the structural refinement of **5**.

Empirical Formula	$C_{66}H_{52}TiP_2S_6$
Formula weight	1147.27 g/mol
Temperature	100(2) K
Wavelength	0.71073 Å
Crystal System	Monoclinic
Space Group	P−1
Unit Cell Dimensions	a = 13.870(4) Å, α = 100.250(5)°
	b = 14.040(4) Å, β = 91.957(5)°
	c = 33.318(10) Å, γ = 110.364(5)°
Volume	5508(3) Å^3
Z	4
Density (calculated)	1.383 Mg/m^3
Absorption coefficient	0.484 mm^{-1}
F_{000}	2384.0
Crystal color	Dark Red
Crystal size	0.18 × 0.17 × 0.12 mm^3
θ range	2.36 to 26.94°
Index ranges	$-17 \leq h \leq 17$
	$-18 \leq k \leq 18$
	$-41 \leq l \leq 43$
Reflections collected	23173
Independent reflections	22963 [R_{int} = 0.0533]
Completeness to θ = 52.48°	96.0 %
Absorption correction	Multi-scan
Maximum and minimum transmission	0.655 and 0.745
Refinement method	Full-matrix least-squares on F^2
Data / restraints / parameters	25013 / 0 / 1353
Goodness-of-fit on F^{2a}	1.176
Final R indices [I > 2σ(I) = 19146 data]^b	R_1 = 12.59 %, wR_2 = 33.70 %
R indices (all data, 0.80 Å)	R_1 = 14.01 %, wR_2 = 33.70 %
Largest diff. peak and hole	1.57 and –0.92 e.Å^{-3}

^{a} GooF = $[\Sigma[w(F_o^2–F_c^2)^2] / (n\text{-}p)]^{1/2}$ where n is the number of reflections and p is the total number of parameters refined. $^b R_1 = \Sigma||F_o|–|F_c|| / \Sigma|F_o|$; $wR_2 = [\Sigma[w(F_o^2–F_c^2)^2] / \Sigma[w(F_o^2)^2]\ ]^{1/2}$

Table S5 | Crystallographic information for the structural refinement of **6**.

Empirical Formula	$C_{60}H_{48}NiP_2S_4$
Formula weight	1117.87 g/mol
Temperature	100(2) K
Wavelength	0.71073 Å
Crystal System	Monoclinic
Space Group	$P2_1/n$
Unit Cell Dimensions	a = 11.1212 (6) Å, α = 90.0°
	b =14.6404(8) Å, β = 95.168(3)°
	c = 314.8691(8) Å, γ = 90.0°
Volume	2411.1(2) Å^3
Z	2
Density (calculated)	1.402 Mg/m^3
Absorption coefficient	0.683 mm^{-1}
F_{000}	1060.0
Crystal color	Dark Red
Crystal size	0.1 × 0.1 × 0.05 mm^3
θ range	1.956 to 30.841°
Index ranges	$-15 \leq h \leq 15$
	$-21 \leq k \leq 21$
	$-21 \leq l \leq 21$
Reflections collected	80663
Independent reflections	78851 [R_{int} = 0.0691]
Completeness to θ = 52.48°	99.4 %
Absorption correction	Multi-scan
Maximum and minimum transmission	0.7021 and 0.7461
Refinement method	Full-matrix least-squares on F^2
Data / restraints / parameters	9898 / 0 / 305
Goodness-of-fit on F^{2a}	1.028
Final R indices [I > 2σ(I) = 10408 data]^b	R_1 = 3.45 %, wR_2 = 6.79 %
R indices (all data, 0.80 Å)	R_1 = 5.61 %, wR_2 = 7.56 %
Largest diff. peak and hole	0.40 and –0.36 e.Å^{-3}

^{a} GooF = $[\Sigma[w(F_o^2-F_c^2)^2] / (n-p)]^{1/2}$ where n is the number of reflections and p is the total number of parameters refined. $^b R_1 = \Sigma||F_o|-|F_c|| / \Sigma|F_o|$; $wR_2 = [\Sigma[w(F_o^2-F_c^2)^2] / \Sigma[w(F_o^2)^2]]^{1/2}$

Table S6 | Crystallographic information for the structural refinement of **7**.

Empirical Formula	$C_{66}H_{52}TiP_2Se_6$
Formula weight	1171.48 g/mol
Temperature	100(2) K
Wavelength	0.71073 Å
Crystal System	Monoclinic
Space Group	*C*2/c
Unit Cell Dimensions	a = 18.3767(16) Å, α = 90.0°
	b = 8.6094(7) Å, β = 100.972(5)°
	c = 31.158(3) Å, γ = 90.0°
Volume	4839.5(8) Å^3
Z	4
Density (calculated)	1.285 Mg/m^3
Absorption coefficient	0.81 mm^{-1}
F_{000}	1211.9
Crystal color	Red
Crystal size	0.1 × 0.1 × 0.07 mm^3
θ range	2.76 to 52.48°
Index ranges	$-11 \leq h \leq 11$
	$-20 \leq k \leq 21$
	$-23 \leq l \leq 21$
Reflections collected	26262
Independent reflections	25142 [R_{int} = 0.0726]
Completeness to θ = 52.48°	94.9 %
Absorption correction	Multi-scan
Maximum and minimum transmission	0.655 and 0.745
Refinement method	Full-matrix least-squares on F^2
Data / restraints / parameters	10963 / 10 / 235
Goodness-of-fit on F^{2a}	1.128
Final R indices [I > 2σ(I) = 10408 data]^b	R_1 = 5.91 %, wR_2 = 10.93 %
R indices (all data, 0.80 Å)	R_1 = 7.91 %, wR_2 = 15.55 %
Largest diff. peak and hole	0.45 and –0.39 e.Å^{-3}

^{a} GooF = $[\Sigma[w(F_o^2-F_c^2)^2] / (n-p)]^{1/2}$ where n is the number of reflections and p is the total number of parameters refined. $^b R_1 = \Sigma||F_o|-|F_c|| / \Sigma|F_o|$; $wR_2 = [\Sigma[w(F_o^2-F_c^2)^2] / \Sigma[w(F_o^2)^2]]^{1/2}$

Table S7 | Crystallographic information for the structural refinement of 8.

Empirical Formula	$C_{60}H_{48}NiP_2Se_4$
Formula weight	1205.07 g/mol
Temperature	100(2) K
Wavelength	0.71073 Å
Crystal System	Monoclinic
Space Group	$C2/c$
Unit Cell Dimensions	a = 11.1660(14) Å, α = 90.0°
	b = 14.8566(19) Å, β = 94.939(2)°
	c = 14.9901 (19) Å, γ = 90.0°
Volume	2477.5(5) Å^3
Z	2
Density (calculated)	1.616 Mg/m^3
Absorption coefficient	3.435 mm^{-1}
F_{000}	1204.0
Crystal color	Dark Red
Crystal size	0.15 × 0.14 × 0.1 mm^3
θ range	1.933 to 29.372°
Index ranges	$-15 \leq h \leq 15$
	$-20 \leq k \leq 20$
	$-20 \leq l \leq 20$
Reflections collected	91325
Independent reflections	88998 [R_{int} = 0.0726]
Completeness to θ = 52.48°	99.0 %
Absorption correction	Multi-scan
Maximum and minimum transmission	0.6615 and 0.7459
Refinement method	Full-matrix least-squares on F^2
Data / restraints / parameters	6782 / 0 / 305
Goodness-of-fit on F^{2a}	1.062
Final R indices [I > 2σ(I) = 5807 data]^b	R_1 = 2.45 %, wR_2 = 5.55 %
R indices (all data, 0.80 Å)	R_1 = 3.46 %, wR_2 = 6.03 %
Largest diff. peak and hole	0.66 and –0.55 e.Å^{-3}

^{a} GooF = $[\Sigma[w(F_o^2–F_c^2)^2] / (n-p)]^{1/2}$ where n is the number of reflections and p is the total number of parameters refined. $^b R_1 = \Sigma||F_o|–|F_c|| / \Sigma|F_o|$; $wR_2 = [\Sigma[w(F_o^2–F_c^2)^2] / \Sigma[w(F_o^2)^2]]^{1/2}$

Table S8 | Select Structural parameters for **1–4**. H_1 and H_2 are the ortho and meta protons of the catechol ligands, respectively.

	**1**	**2**	**3**	**4**
M–E	2.372(11)	2.487(13)	2.279(2)	2.416(25)
M···H_1	5.088(12)	5.241(2)	4.923(5)	5.119(9)
M···H_2	6.639(8)	6.812(15)	6.457(8)	6.664(1)
M···M	10.935(2)	10.733(1)	11.136(6)	11.684(4)

Table S9 | Variable-temperature Cole-Cole plot fit parameters for **1** under 3400 Oe dc applied field.

**Temperature (K)**	χ_T **(cm^3/mol)**	χ_S **(cm^3/mol)**	τ **(ms)**	α
5	0.307	0	37.7	0.249
7.5	0.198	0	18.6	0.24
10	0.14	0	13	0.15
12.5	0.11	0	7	0.1
15	0.09	0	4	0
17.5	0.08	0	2	0
20	0.065	0	1.2	0
22.5	0.06	0	0.7	0
25	0.005	0	0.5	0

Table S10 | Variable-temperature Cole-Cole plot fit parameters for **2** under 3400 Oe dc applied field.

**Temperature (K)**	χ_T **(cm^3/mol)**	χ_S **(cm^3/mol)**	τ **(ms)**	α
5	0.262	0	137.5	0.03
7.5	0.172	0	47.2	0.003
10	0.129	0	17.6	0.01
12.5	0.101	0	7.7	0.005
15	0.08	0	4	0
17.5	0.072	0	2.3	0
20	0.061	0	1.5	0
22.5	0.055	0	1.1	0
25	0.048	0	0.78	0

Table S11 | Variable-temperature Cole-Cole plot fit parameters for **3** under 3400 Oe dc applied field.

**Temperature (K)**	**χ_T (cm^3/mol)**	**χ_S (cm^3/mol)**	**τ (ms)**	**α**
5	0.25	0	3.5	0.2
7.5	0.16	0	2	0.15
10	0.115	0	1.3	0.1
12.5	0.09	0	0.8	0.1
15	0.073	0	0.5	0
17.5	0.062	0	0.3	0
20	0.053	0	0.2	0
22.5	0.045	0	0.12	0
25	0.04	0	0.09	0

Table S12 | Variable-temperature Cole-Cole plot fit parameters for **4** under 3400 Oe dc applied field.

**Temperature (K)**	**χ_T (cm^3/mol)**	**χ_S (cm^3/mol)**	**τ (ms)**	**α**
5	0.296	0	33.9	0.28
7.5	0.194	0	10.4	0
10	0.145	0	3.75	0.002
12.5	0.116	0	1.55	0.004
15	0.095	0	0.78	0
17.5	0.08	0	0.45	0
20	0.07	0	0.31	0
22.5	0.064	0	0.22	0
25	0.055	0	0.18	0

Table S13 | Variable-field Cole-Cole plot fit parameters for **1** at 5 K.

**Field (Oe)**	χ_T **(cm^3/mol)**	χ_S **(cm^3/mol)**	τ **(ms)**	α
250	0.299	0.198	3.59	0.438
500	0.301	0.111	7.56	0.233
750	0.306	0.055	9.6	0.256
1000	0.289	0	11.8	0.288
2000	0.297	0.008	30.0	0.179
3000	0.306	0	40.6	0.246
4000	0.302	0	45.6	0.242
5000	0.303	0	50.7	0.259
6000	0.302	0	53.9	0.254
7000	0.300	0	55.6	0.261
8000	0.297	0	55.1	0.260
9000	0.298	0	55.3	0.260
10000	0.298	0	54.3	0.259
12000	0.293	0	52.5	0.252
14000	0.287	0	47.5	0.23
16000	0.290	0	41.8	0.245
18000	0.279	0	37.7	0.224
20000	0.279	0	29.0	0.231
25000	0.268	0	20.2	0.190
30000	0.264	0	13.48	0.129
35000	0.254	0	9.74	0.171

Table S14 | Variable-field Cole-Cole plot fitting parameters for **2** at 5 K.

**Field (Oe)**	χ_T **(cm^3/mol)**	χ_S **(cm^3/mol)**	τ **(ms)**	α
250	0.265	0.113	7.02	0.09
500	0.265	0.037	12.3	0.07
750	0.266	0.012	19.2	0.08
1000	0.264	0.001	28.7	0.07
2000	0.263	0	77.8	0.04
3000	0.263	0	122.3	0.03
4000	0.261	0	158.8	0.02
5000	0.262	0	183.6	0.03
6000	0.258	0	199.9	0.02
7000	0.259	0	203.2	0.02
8000	0.258	0	199.9	0.02
9000	0.253	0	186.4	0.006
10000	0.253	0	174.0	0.01
12000	0.248	0	135.7	0.009
14000	0.244	0	99.4	0.01
16000	0.241	0	70.5	0.03
18000	0.236	0	49.8	0.02
20000	0.228	0	36.4	0
25000	0.211	0	15.4	0
30000	0.204	0	7.42	0
35000	0.194	0	4.84	0

Table S15 | Variable-field Cole-Cole plot fit parameters for **3** at 5 K

Field (Oe)	χ_T (cm^3/mol)	χ_S (cm^3/mol)	τ (ms)	α
250	0.253	0.169	0.091	0.228
500	0.254	0.094	1.28	0.170
750	0.255	0.055	1.56	0.175
1000	0.257	0.035	1.84	0.180
2000	0.260	0.007	2.49	0.202
3000	0.259	0.003	2.72	0.199
4000	0.259	0	2.93	0.205
5000	0.258	0	3.11	0.206
6000	0.259	0	3.17	0.213
7000	0.257	0	3.26	0.211
8000	0.379	0	3.27	0.211
9000	0.256	0	3.30	0.212
10000	0.254	0	3.32	0.206
12000	0.250	0	3.33	0.205
14000	0.250	0	3.39	0.223
16000	0.246	0	3.44	0.222
18000	0.240	0	3.37	0.220
20000	0.239	0	3.26	0.237
25000	0.228	0	3.02	0.219
30000	0.218	0	2.67	0.235
35000	0.208	0	2.23	0.219

Table S16 | Variable-field Cole-Cole plot fitting parameters for **4** at 5 K.

Field (Oe)	χ_T (cm^3/mol)	χ_S (cm^3/mol)	τ (ms)	α
250	0.305	0.13	2.54	0.25
500	0.305	0.05	5.05	0.17
750	0.305	0.02	8.03	0.14
1000	0.304	0	10.9	0.12
2000	0.301	0	22.5	0.07
3000	0.303	0	33.5	0.06
4000	0.299	0	41.0	0.03
5000	0.299	0	48.0	0.03
6000	0.298	0	54.5	0.04
7000	0.298	0	53.3	0.04
8000	0.295	0	52.9	0.03
9000	0.294	0	49.9	0.04
10000	0.291	0	46.1	0.02
12000	0.287	0	34.7	0.03
14000	0.281	0	25.9	0.03
16000	0.278	0	18.4	0.05
18000	0.269	0	14.1	0.01
20000	0.263	0	9.96	0.02
25000	0.255	0	4.34	0
30000	0.241	0.001	2.67	0.06
35000	0.212	0.006	1.36	0.02

Table S17 | Saturation recovery fit parameters for **1′**.

**Temperature (K)**	T_1 **(ms)**	S **(ms)**	A	I_0
5	45.0	785	1.00	0.0
10	25.5	557	0.99	0.0
15	5.83	360	0.99	0.0
20	1.73	359	0.99	0.0
30	0.26	158	0.99	0.0
40	6.51×10^{-2}	25	0.99	0.0
50	2.42×10^{-2}	269	0.99	0.0
60	1.24×10^{-2}	-	0.99	0.0
70	6.58×10^{-3}	-	1.01	0.0
80	4.21×10^{-3}	-	1.02	0.0
90	2.67×10^{-3}	-	1.03	0.0
100	1.93×10^{-3}	-	1.03	0.0

Table S18 | Saturation recovery fit parameters for **2′**.

**Temperature (K)**	T_1 **(ms)**	S **(ms)**	A	I_0
5	30.7	272	0.98	0.0
10	26.7	161	0.99	0.0
15	7.42	43.0	0.99	0.0
20	2.59	51.9	0.99	0.01
30	0.73	15.6	0.98	0.01
40	0.35	2.7	0.98	0.01
50	0.21	0.65	1.00	0.0
60	0.13	0.34	0.99	−0.01
70	0.10	0.15	1.01	−0.02
80	5.05×10^{-2}	8.8×10^{-2}	1.03	−0.04
90	3.48×10^{-2}	7.4×10^{-2}	1.04	−0.04
100	2.49×10^{-2}	6.6×10^{-3}	1.05	−0.05
110	1.74×10^{-2}	6.9×10^{-3}	1.05	−0.05
120	1.17×10^{-2}	9.8×10^{-3}	1.04	−0.05
130	8.71×10^{-3}	0.14	1.05	−0.05
140	6.97×10^{-3}	0.15	1.03	−0.03
160	5.52×10^{-3}	0.64	1.02	−0.02
180	3.61×10^{-3}	1.11	1.03	−0.04
200	2.63×10^{-3}	-	1.04	−0.04
240	1.93×10^{-3}	-	1.06	−0.07
280	1.25×10^{-3}	-	1.07	−0.07

Table S19 | Saturation recovery fit parameters for **3′**.

**Temperature (K)**	T_1 **(ms)**	S **(s)**	A	I_0
5	205	1.09	0.93	0.03
10	6.68	4.16	0.99	0.0
15	1.76	4.67	0.99	0.0
20	0.31	0.22	0.98	0.0
30	4.91×10^{-2}	-	1.00	0.0
40	1.61×10^{-2}	-	0.99	0.0
50	1.13×10^{-2}	-	0.98	−0.01
60	3.43×10^{-3}	-	1.01	−0.02
70	2.75×10^{-3}	-	0.95	−0.04
80	1.36×10^{-3}	-	1.06	−0.06
90	1.39×10^{-3}	-	0.99	−0.06
100	6.90×10^{-4}	-	1.1	−0.10

Table S20 | Saturation recovery fit parameters for **4′**.

**Temperature (K)**	T_1 **(ms)**	S **(ms)**	A	I_0
5	91.4	1201	1.00	0.0
10	12.9	640	1.00	0.0
15	1.94	339	1.00	0.0
20	0.63	197	0.99	0.0
30	0.15	6.84	1.00	0.0
40	4.15×10^{-2}	13.3	0.99	0.0
50	1.71×10^{-2}	0.60	1.01	−0.02
60	1.03×10^{-2}	0.98	0.99	−0.02
70	7.02×10^{-3}	0.16	1.03	−0.04
80	6.72×10^{-3}	0.13	1.01	−0.04
90	5.72×10^{-3}	3.83×10^{-2}	1.03	−0.07
100	3.55×10^{-3}	0.13	1.05	−0.05
110	3.13×10^{-3}	0.17	1.00	−0.04
120	2.81×10^{-3}	-	1.00	−0.02
130	2.25×10^{-3}	-	1.00	−0.03
140	1.49×10^{-3}	-	1.02	−0.05
160	1.15×10^{-3}	-	1.04	−0.07
180	7.66×10^{-4}	-	1.08	−0.10
200	6.92×10^{-4}	-	1.11	−0.12
220	4.69×10^{-4}	-	1.19	−0.18
240	3.78×10^{-4}	-	1.33	−0.26

Table S21 | Two-pulse Hahn-echo dephasing (T_2) fit parameters for **1′–4′**, in units of ns.

Temperature (K)	1′	2′		3′	4′	
5	2156 ± 14	2048 ± 69	652 ± 70	3820 ± 80	3183 ± 61	444 ± 30
10	2572 ± 16	2226 ± 81	714 ± 98	4080 ± 14	4396 ± 701	1484 ± 418
15	2872 ± 16	2233 ± 213	1036 ± 212	5060 ± 94	5381 ± 736	2382 ± 287
20	2916 ± 16	2473 ± 12		3554 ± 28	3018 ± 24	
30	2812 ± 15	1914 ± 8		3165 ± 16	2405 ± 17	
40	2560 ± 14	1634 ± 9		2288 ± 9	1815 ± 12	
50	2208 ± 12	1456 ± 7		1664 ± 26	1447 ± 6	
60	1766 ± 10	1137 ± 5		1256 ± 8	1397 ± 9	
70	1438 ± 9	1129 ± 7		828 ± 19	987 ± 4	
80	1118 ± 7	982 ± 9		724 ± 10	969 ± 11	
90	916 ± 6	903 ± 8		546 ± 20	781 ± 9	
100	742 ± 3	831 ± 8		523 ± 14	816 ± 9	
110	-	784 ± 8		-	653 ± 7	
120	-	722 ± 9		-	638 ± 7	
130	-	708 ± 9		-	578 ± 6	
140	-	693 ± 8		-	485 ± 5	
160	-	683 ± 8		-	419 ± 4	
180	-	692 ± 8		-	356 ± 4	
200	-	653 ± 6		-	324 ± 4	
240	-	597 ± 5		-	228 ± 3	
280	-	521 ± 5		-	194 ± 3	

Table S22 | Fit parameters for the field dependence of τ in **1–4**.

**Temperature (K)**	**1**	**2**	**3**	**4**
c ($T^{-4}ms^{-1}$)	0.31 ± 0.01	1.58 ± 0.2	0.35 ± 0.04	5.98 ± 0.3
d (ms^{-1})	73.1 ± 17	158.6 ± 0.2	311.8 ± 22	253.2 ± 0.4
e (T^{-2})	15.2 ± 1.4	9.2 ± 0.2	39.2 ± 5.4	9.2 ± 0.8
f (T^{-2})	212.5 ± 62	389.2 ± 50	122.6 ± 23	162 ± 30

Table S23 | M-E bond distances for the DFT optimized geometries, compared with experimental distances. Distances are in units of Å.

Species	Exp.	M06-L (gas)	M06-L (DMF)	B3LYP (DMF)
**1**	2.372	2.399	2.395	2.401
**2**	2.487	2.505	2.536	2.539
**3**	2.279	2.336	2.326	2.336
**4**	2.416	2.461	2.470	2.482

Table S24 | NBO spin densities at the metal center.

Species	M06-L (gas)	M06-L (DMF)	B3LYP (DMF)
**1**	1.455	1.444	1.339
**2**	1.579	1.564	1.421
**3**	0.352	0.347	0.335
**4**	0.269	0.292	0.302

Table S25 | MS-CASPT2 ***g***-tensors for structures **1–4.**

	**1**	**2**	**3**	**4**
$g_{\parallel}$	2.002	2.233	2.000	2.245
$g_{\perp}$	1.971	2.053	1.943	2.059
	1.971	2.055	1.952	2.062

Table S26 | Tabulated intramolecular vibrational modes of **1**–**4** computed using the M06-L functional. Energies are in units of cm^{-1}.

**1**	**2**	**3**	**4**
5.97	9.89	7.17	25.22
23.06	39.52	8.38	50.31
32.62	58.09	23.16	52.44
58.22	80.76	39.58	68.38
61.99	123.89	42.81	77.18
70.66	126.48	56.6	97
112.18	139.87	71.47	102.01
119.07	141.57	73.08	105.13
125.6	152.72	84.41	127.26
140.68	208.72	107.21	152.48
143.03	234.7	112.78	180.03
151.03	237.47	114.19	185.74
163.6	245.88	118.73	197.46
173.6	307.06	122.02	212.49
174.6	307.57	125.56	217.7
236.88	348.47	169.15	240.86
239.42	375.61	170.66	265.99
245.12	442.45	180.91	364
269.68	443.25	207.26	364.25
275.97	447.96	207.83	370.53
276.74	458.34	211.78	371.69
317.14	486.63	224.3	443.88
324.46	492.72	242.41	445.1
327.47	538.06	244.4	508.05
367.61	538.62	260.48	509.63
370.57	670.87	269.31	652.92
391.78		274.04	
446.39		362.93	
447.35		363.43	
448.6		365.11	
456.5		371.89	
457.5		372.72	
460.41		375.1	
489.56		435.3	
490.05		436.48	
491.75		437.6	
537.18		515.23	
537.55		516.56	
538.15		517.2	
678.38		655.73	

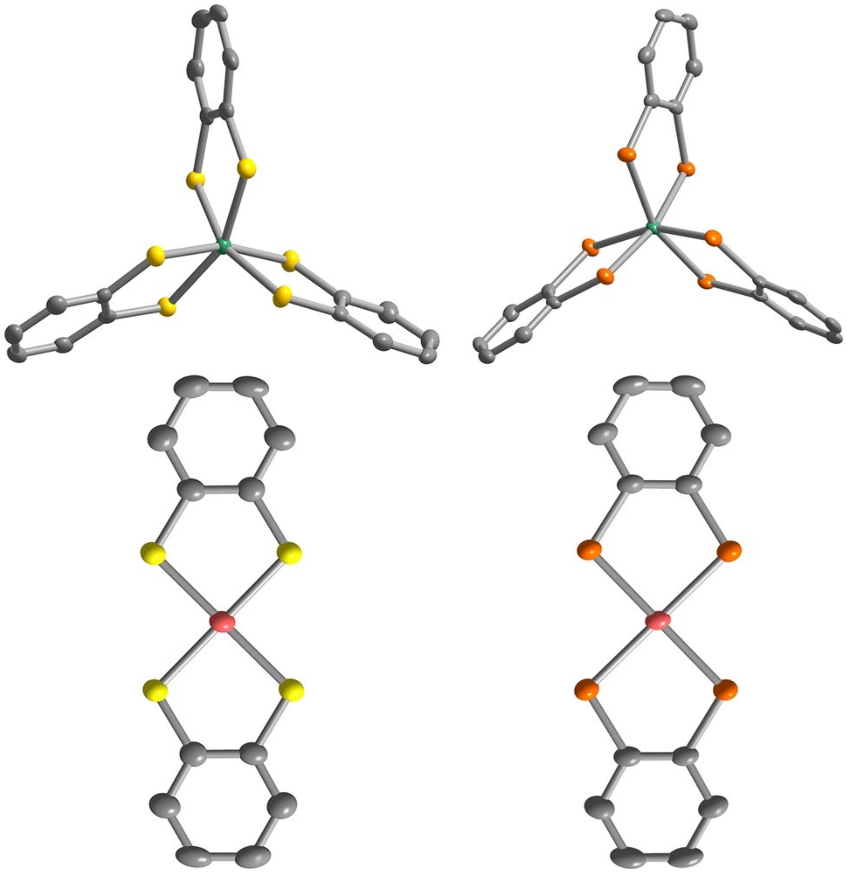

Figure S1 | Thermal ellipsoid plots of the vanadium and copper complexes in the crystal structures of **1**–**4**, depicted at the 70 % probability level.

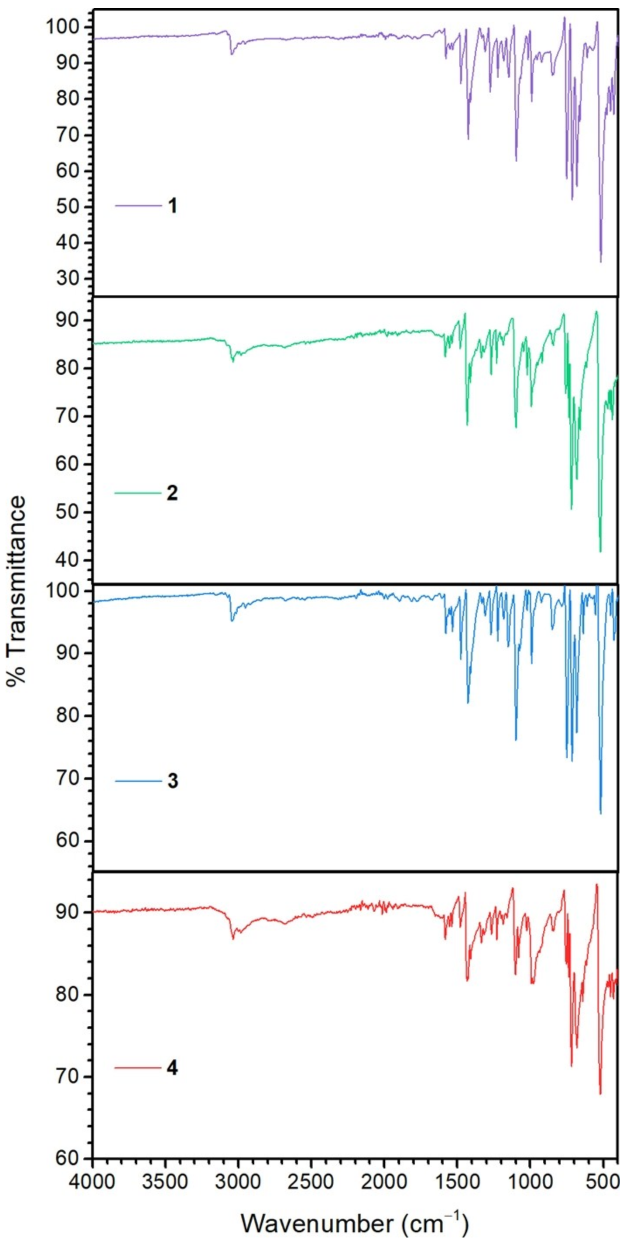

Figure S2 | Infrared spectroscopic data on **1**–**4**. All data were collected on solid samples at room temperature under a dinitrogen atmosphere.

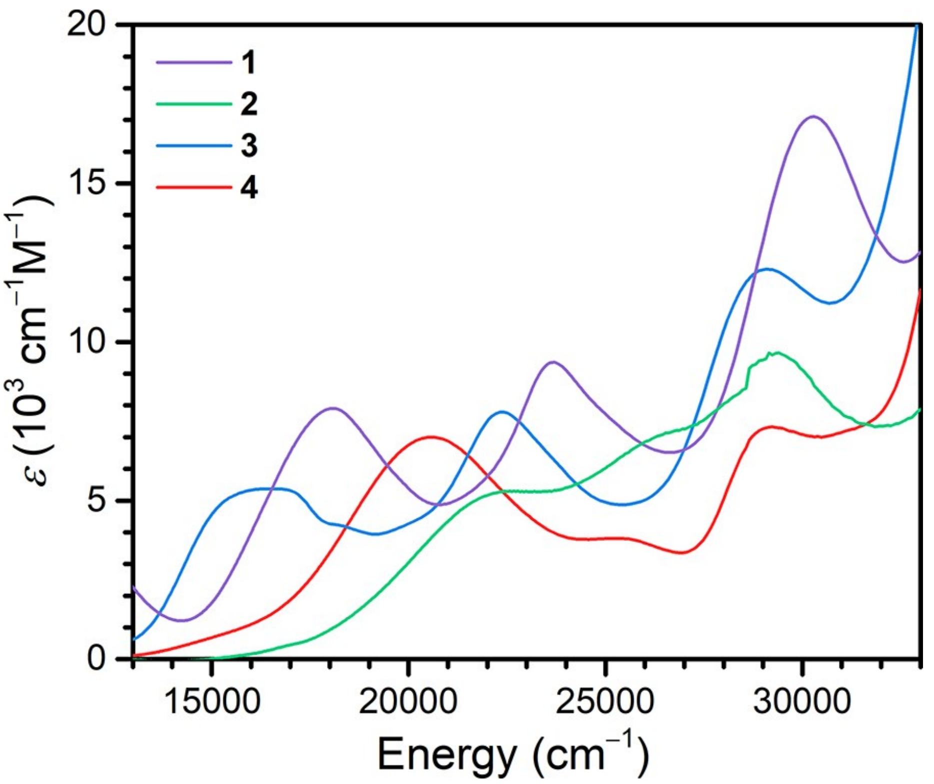

Figure S3 | Electronic absorption (UV-Vis) spectra for **1–4.** Spectra were collected at room temperature in either MeCN (**1** and **3**) or DMF (**2** and **4**) solution.

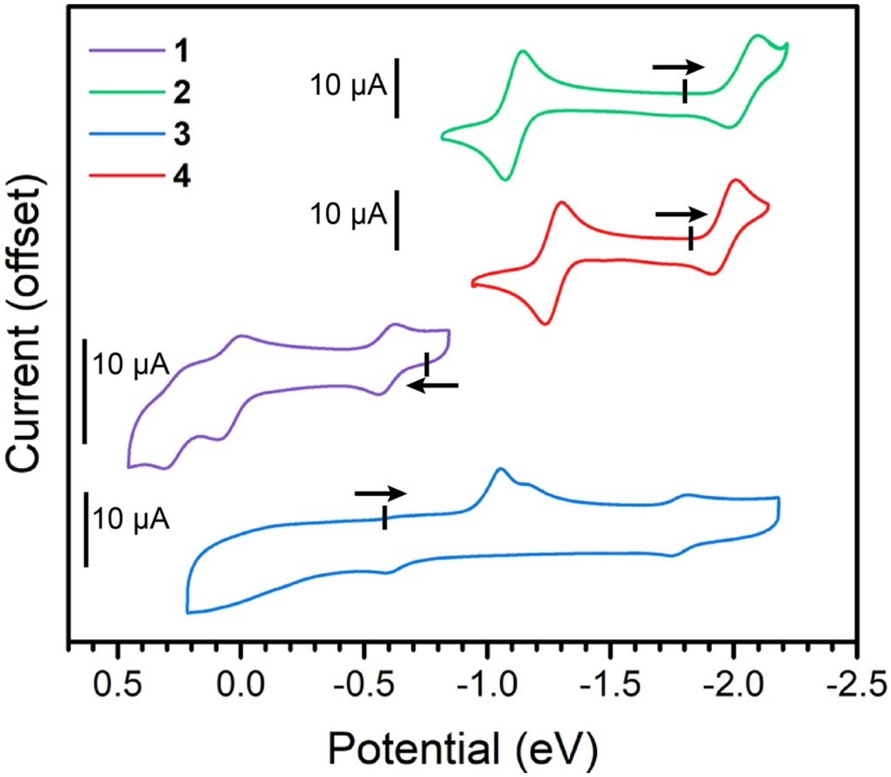

Figure S4 | Solution cyclic voltammetry data for **1**–**4**. Data were collected at room temperature in acetonitrile for **1** and **3**, and in DMF for **2** and **4**, using 0.1 M $(Bu_4N)[PF_6]$ as a supporting electrolyte. Data are normalized for clarity; black vertical bars on the left side of the plot represent 10 µA current for each sample. Small vertical black bars on the cyclic voltammograms indicate starting potentials for the measurements and arrows indicate the starting direction of the measurement.

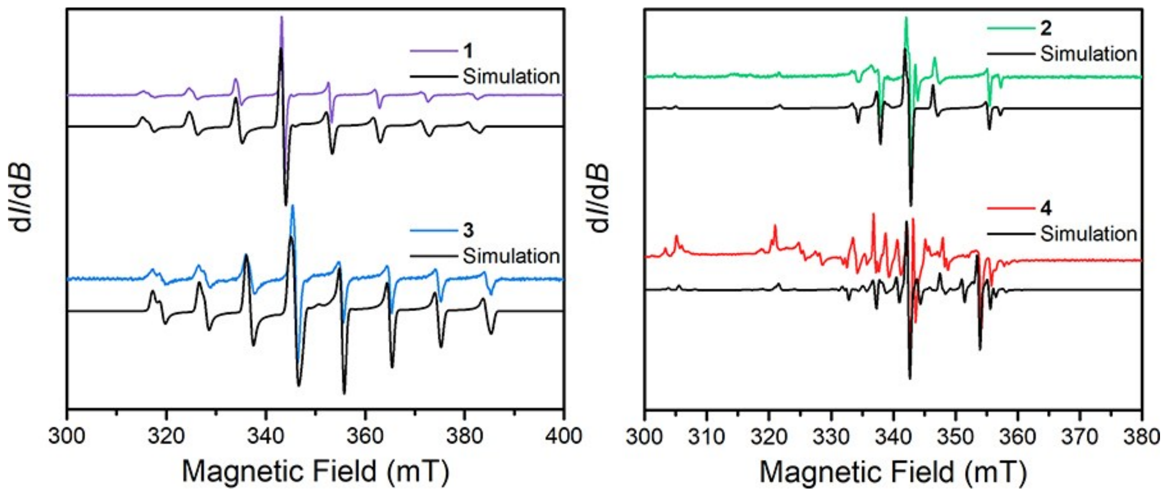

Figure S5 | Overlay of cw EPR spectra collected on **1′–4′** at 20 K. See main manuscript for Hamiltonian and fit parameters.

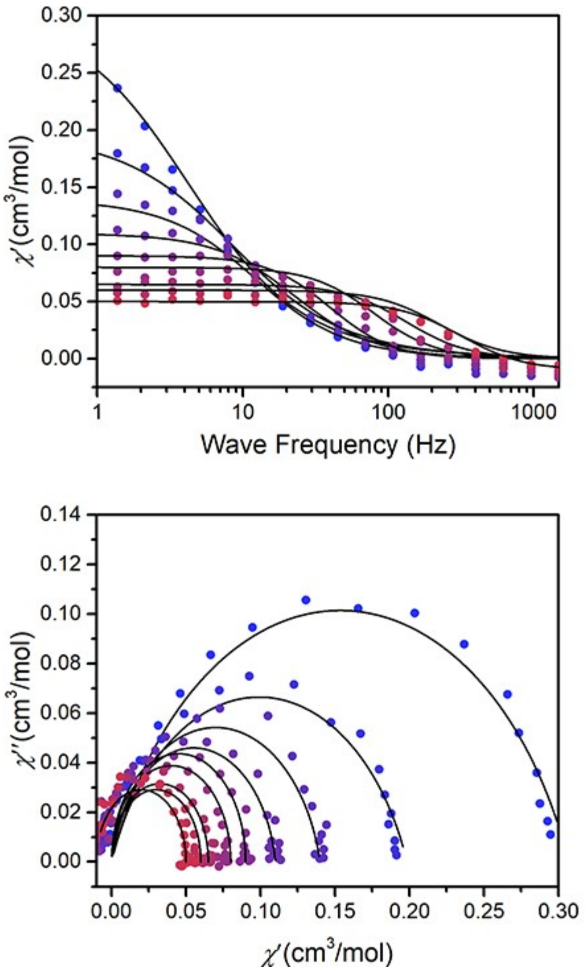

Figure S6 | Variable-temperature ac magnetic susceptibility of **1**. Top-left: in-phase ac magnetic susceptibility of **1**. Top-right: out-of-phase ac magnetic susceptibility of **1**. Bottom-left: Cole-Cole plots generated for **1**. Bottom-right: temperature dependence of τ. Data were collected at 3400 G in the temperature range of 5 K to 25 K, in 2.5 K increments. All black lines are fits to the data using the generalized Debye model.

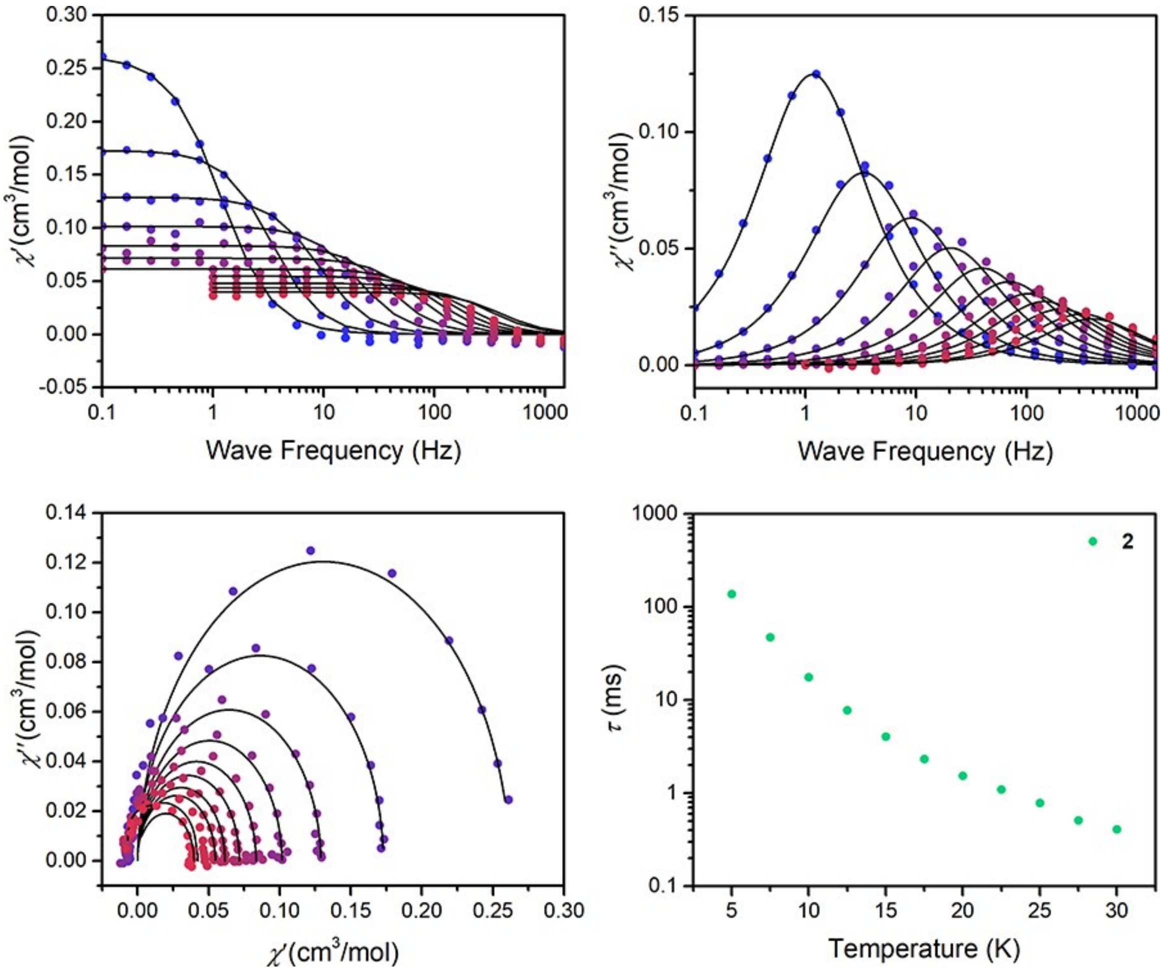

Figure S7 | Variable-temperature ac magnetic susceptibility of **2**. Top-left: in-phase ac magnetic susceptibility of **2**. Top-right: out-of-phase ac magnetic susceptibility of **2**. Bottom-left: Cole-Cole plots generated for **2**. Bottom-right: temperature dependence of τ. Data were collected at 3400 G in the temperature range of 5 K to 25 K, in 2.5 K increments. All black lines are fits to the data using the generalized Debye model.

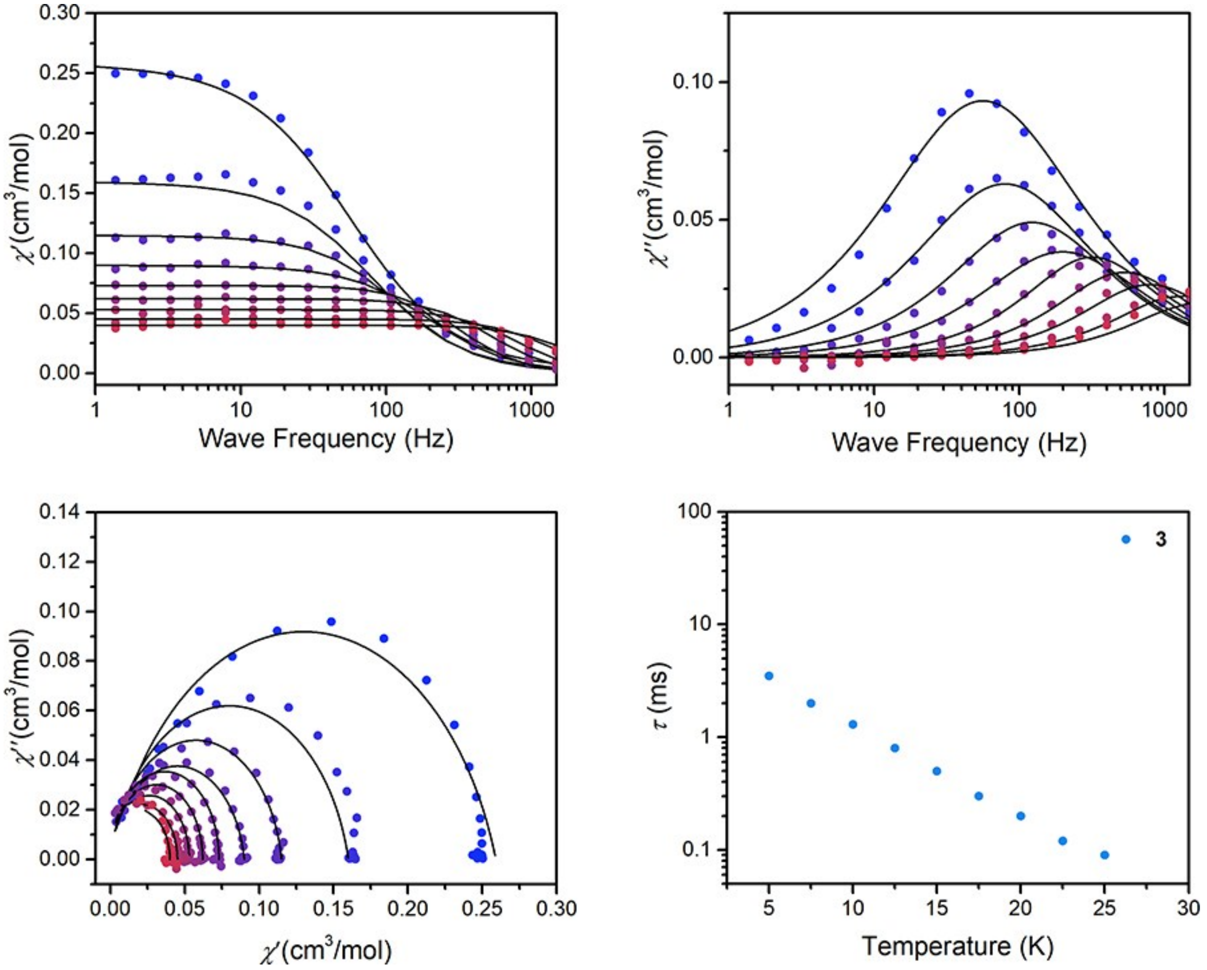

Figure S8 | Variable-temperature ac magnetic susceptibility of **3**. Top-left: in-phase ac magnetic susceptibility of **3**. Top-right: out-of-phase ac magnetic susceptibility of **3**. Bottom-left: Cole-Cole plots generated for **3**. Bottom-right: temperature dependence of τ. Data were collected at 3400 G in the temperature range of 5 K to 25 K, in 2.5 K increments. All black lines are fits to the data using the generalized Debye model.

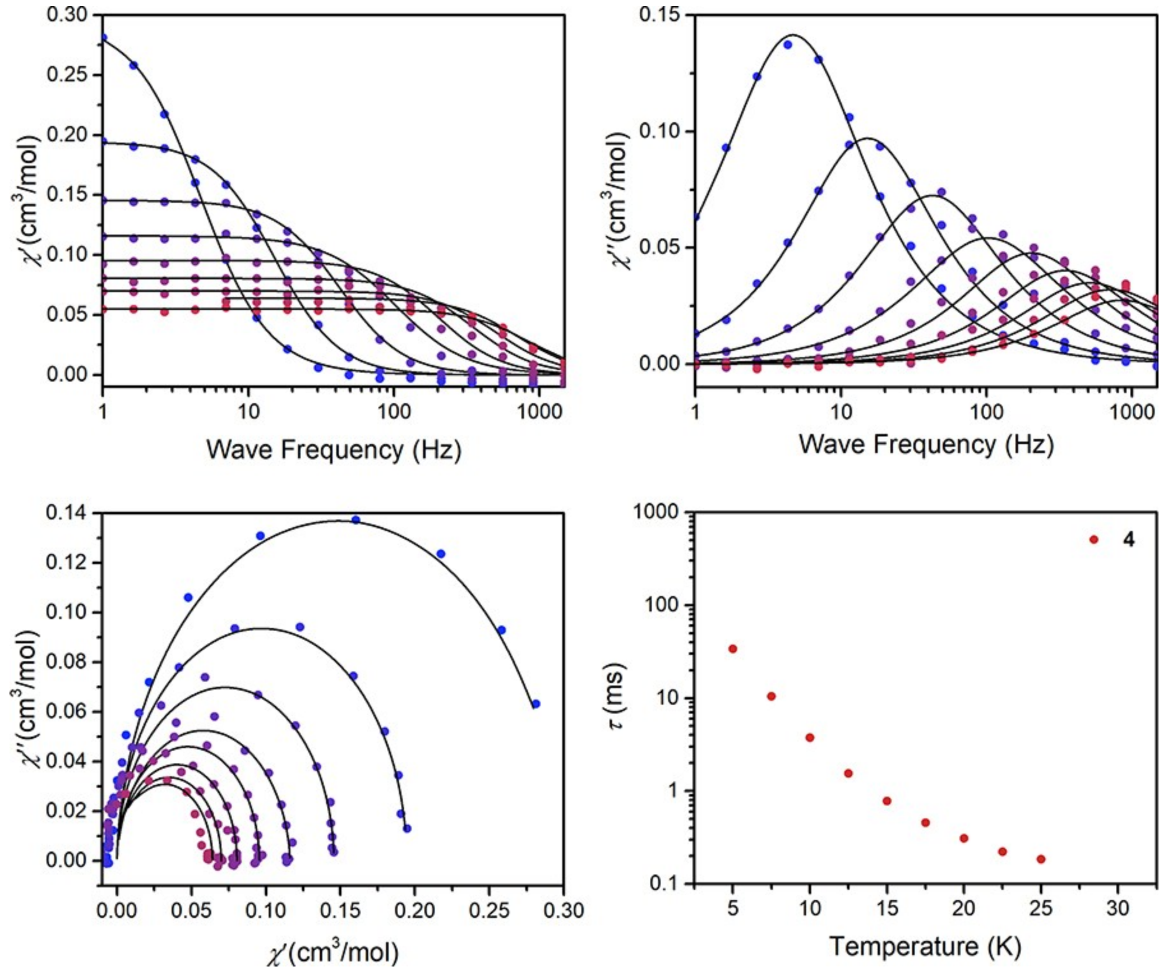

Figure S9 | Variable-temperature ac magnetic susceptibility of **4**. Top-left: in-phase ac magnetic susceptibility of **4**. Top-right: out-of-phase ac magnetic susceptibility of **4**. Bottom-left: Cole-Cole plots generated for **4**. Bottom-right: temperature dependence of τ. Data were collected at 3400 G in the temperature range of 5 K to 25 K, in 2.5 K increments. All black lines are fits to the data using the generalized Debye model.

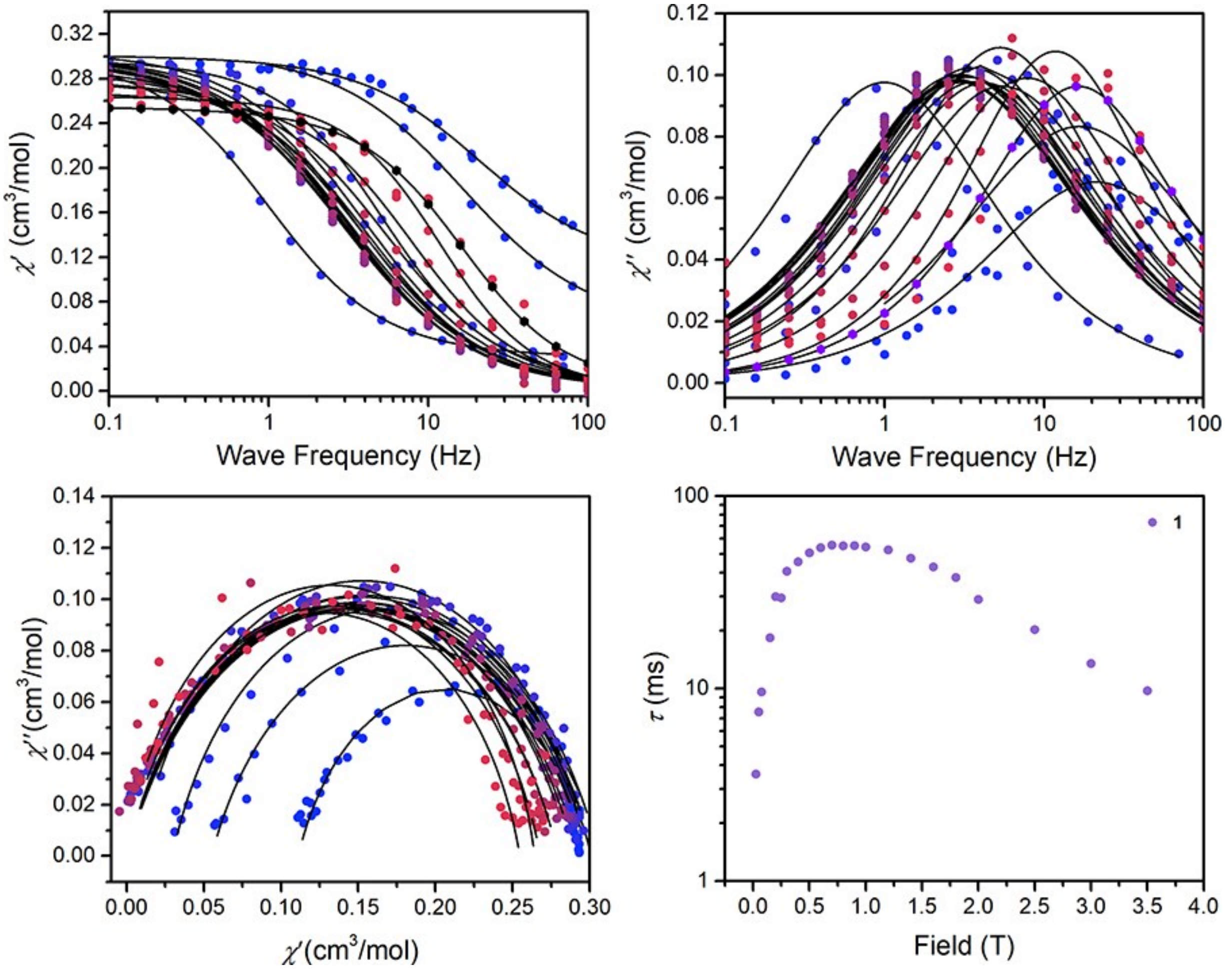

Figure S10 | Variable-field ac magnetic susceptibility of **1**. Top-left: variable-field in-phase ac magnetic susceptibility of **1**. Top-right: variable-field out-of-phase ac magnetic susceptibility of **1**. Bottom-left: variable-field Cole-Cole plots generated for **1**. Bottom-right: magnetic field dependence of τ. Data were collected at 5 K in the field range of 250 Oe to 35000 Oe (or 3.5 T). All black lines are fits to the data using the generalized Debye model.

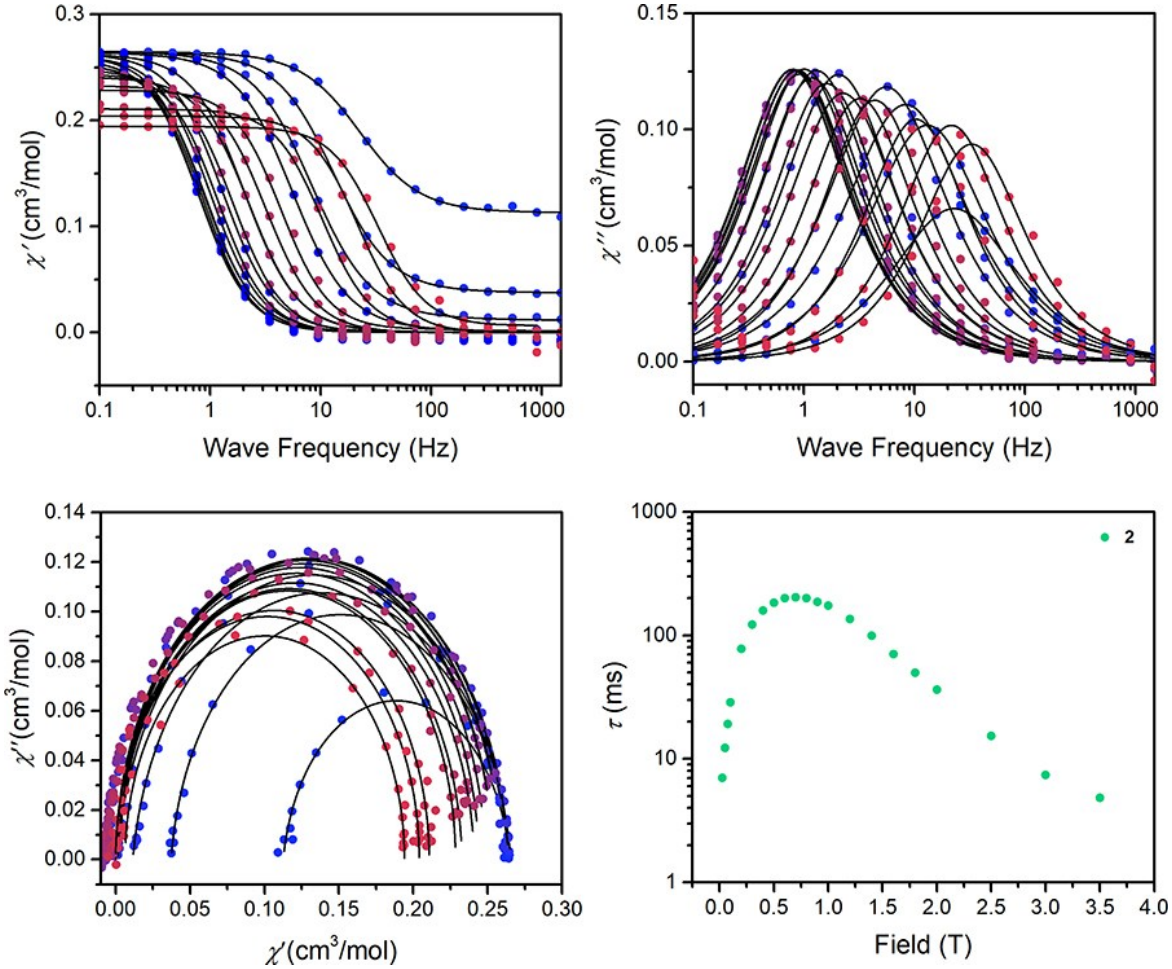

Figure S11 | Variable-field ac magnetic susceptibility of **2**. Top-left: variable-field in-phase ac magnetic susceptibility of **2**. Top-right: variable-field out-of-phase ac magnetic susceptibility of **2**. Bottom-left: variable-field Cole-Cole plots generated for **2**. Bottom-right: magnetic field dependence of τ. Data were collected at 5 K in the field range of 250 Oe to 35000 Oe (or 3.5 T). All black lines are fits to the data using the generalized Debye model.

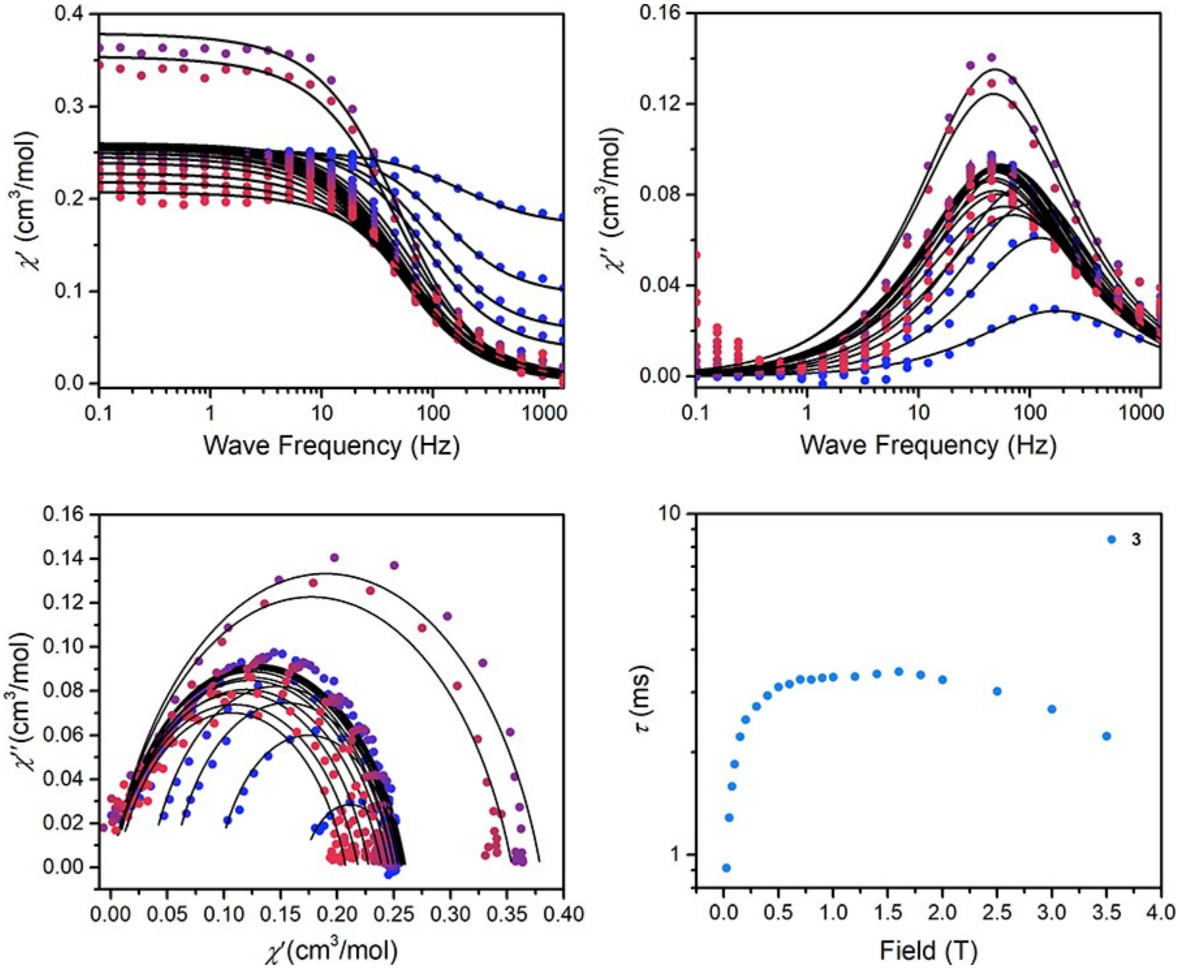

Figure S12 | Variable-field ac magnetic susceptibility of **3**. Top-left: variable-field in-phase ac magnetic susceptibility of **3**. Top-right: variable-field out-of-phase ac magnetic susceptibility of **3**. Bottom-left: variable-field Cole-Cole plots generated for **3**. Bottom-right: magnetic field dependence of τ. Data were collected at 5 K in the field range of 250 Oe to 35000 Oe (or 3.5 T). All black lines are fits to the data using the generalized Debye model.

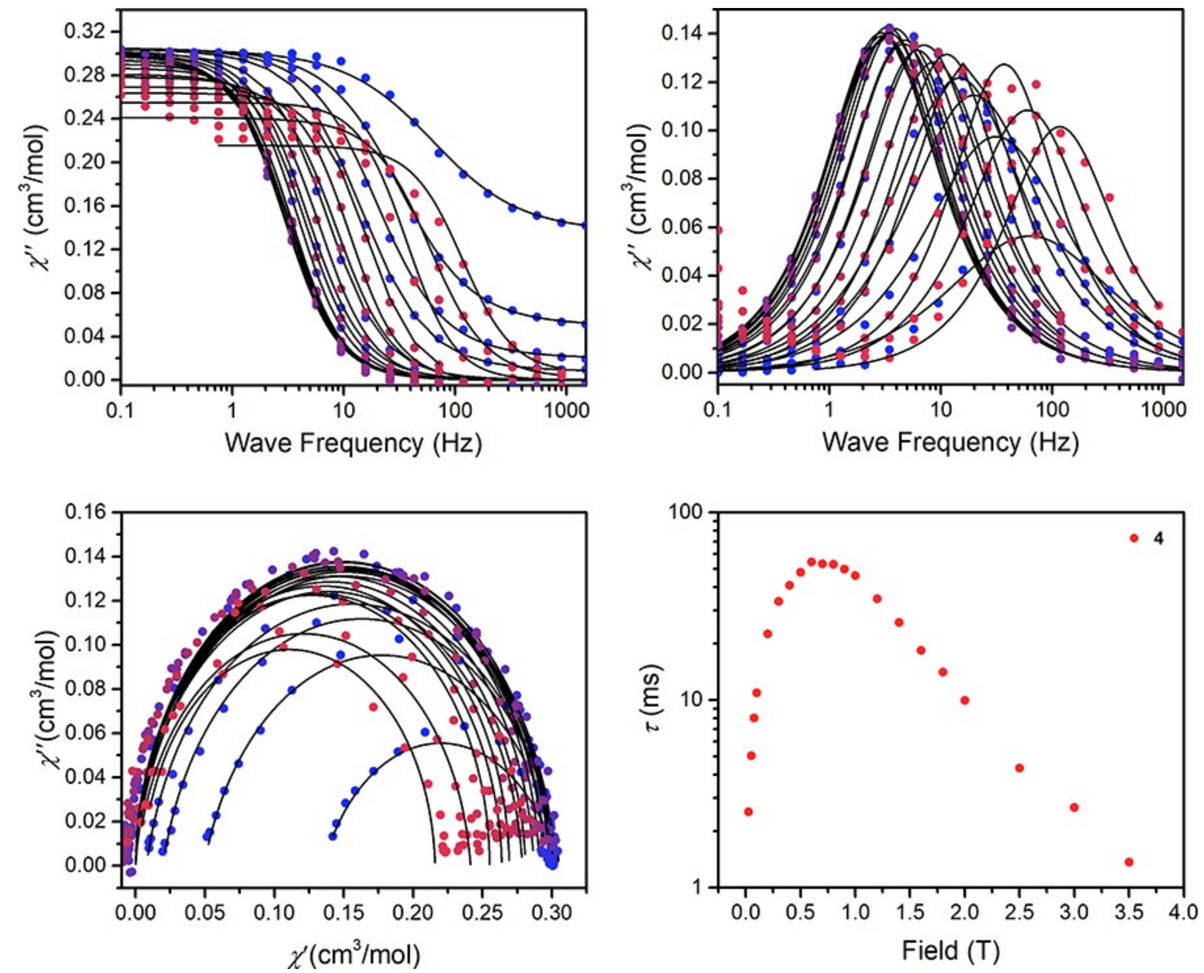

Figure S13 | Variable-field ac magnetic susceptibility of **4**. Top-left: variable-field in-phase ac magnetic susceptibility of **4**. Top-right: variable-field out-of-phase ac magnetic susceptibility of **4**. Bottom-left: variable-field Cole-Cole plots generated for **4**. Bottom-right: magnetic field dependence of *τ*. Data were collected at 5 K in the field range of 250 Oe to 35000 Oe (or 3.5 T). All black lines are fits to the data using the generalized Debye model.

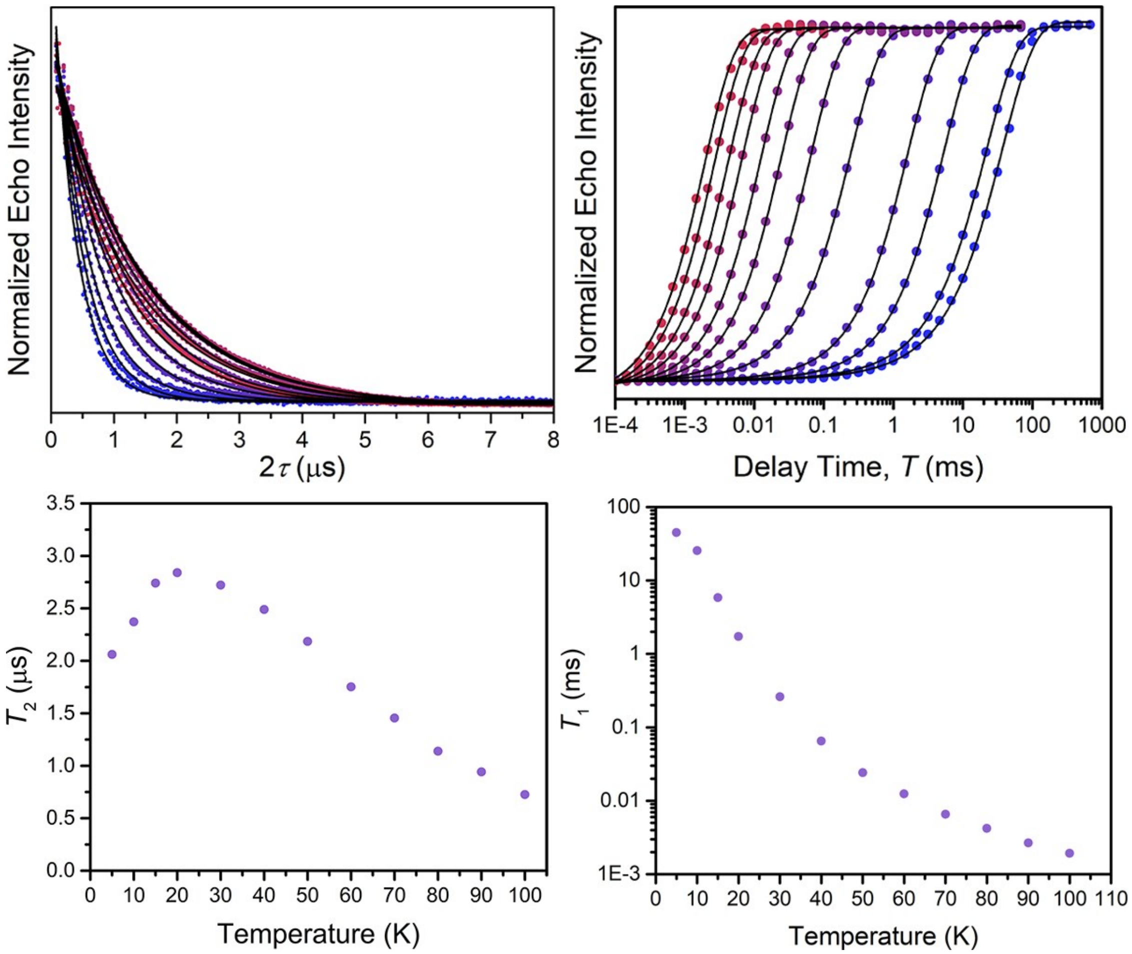

Figure S14 | Pulsed X-band EPR measurements of **1′**. Top-left: variable-temperature T_2 decay curves, collected through application of a Hahn-echo pulse sequence. Black lines are best fits to the data using a mono-exponential decay function. Top-right: saturation recovery curves for **1′** collected through application of twenty consecutive inversion pulses, followed by a Hahn-echo sequence for detection. Black lines are best fits to the data using the decay function described above to account for spectral diffusion. Bottom-left: temperature dependence of T_2 for **1′**. Bottom-right: temperature dependence of T_1 for **1′**. Data were collected from 5 K to 100 K, beyond which an echo could no longer be observed.

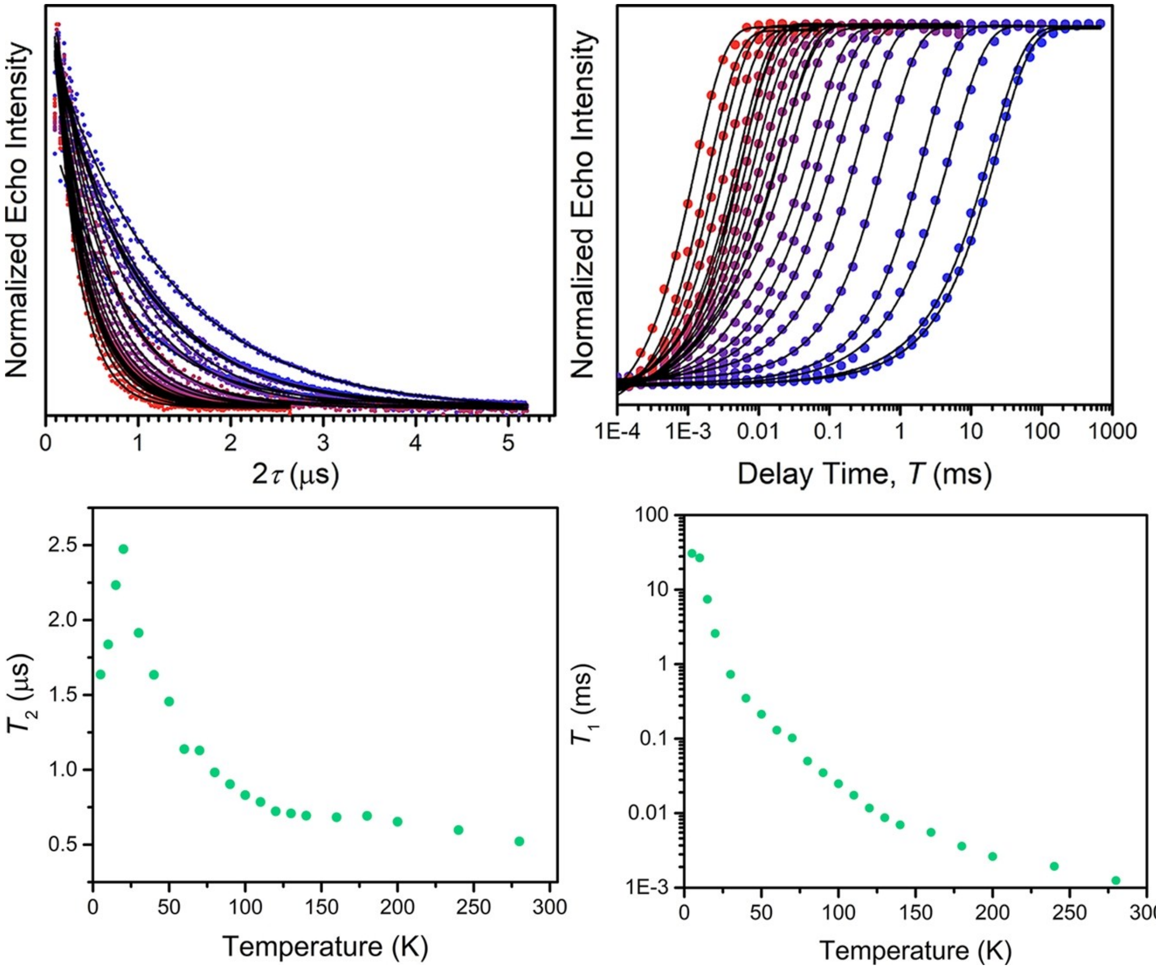

Figure S15 | Pulsed X-band EPR measurements of **2′**. Top-left: variable-temperature T_2 decay curves, collected through application of a Hahn-echo pulse sequence. Black lines are best fits to the data using a mono-exponential decay function. Top-right: saturation recovery curves for **2′** collected through application of twenty consecutive inversion pulses, followed by a Hahn-echo sequence for detection. Black lines are best fits to the data using the decay function described above to account for spectral diffusion. Bottom-left: temperature dependence of T_2 for **2′**. Bottom-right: temperature dependence of T_1 for **2′**. Data were collected from 5 K to 280 K.

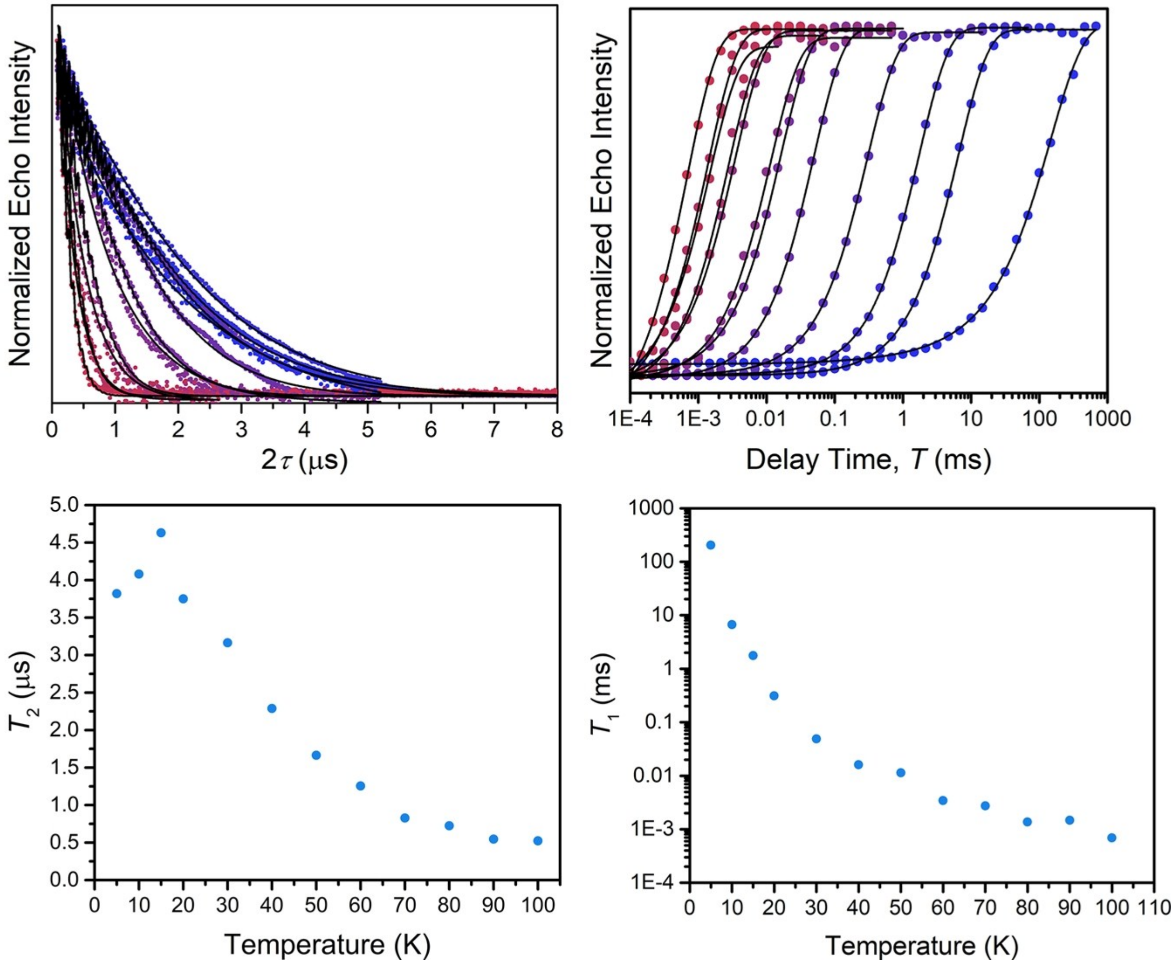

Figure S16 | Pulsed X-band EPR measurements of **3′**. Top-left: variable-temperature T_2 decay curves, collected through application of a Hahn-echo pulse sequence. Black lines are best fits to the data using a mono-exponential decay function. Top-right: saturation recovery curves for **3′** collected through application of twenty consecutive inversion pulses, followed by a Hahn-echo sequence for detection. Black lines are best fits to the data using the decay function described above to account for spectral diffusion. Bottom-left: temperature dependence of T_2 for **3′**. Bottom-right: temperature dependence of T_1 for **3′**. Data were collected from 5 K to 100 K, beyond which an echo could no longer be detected.

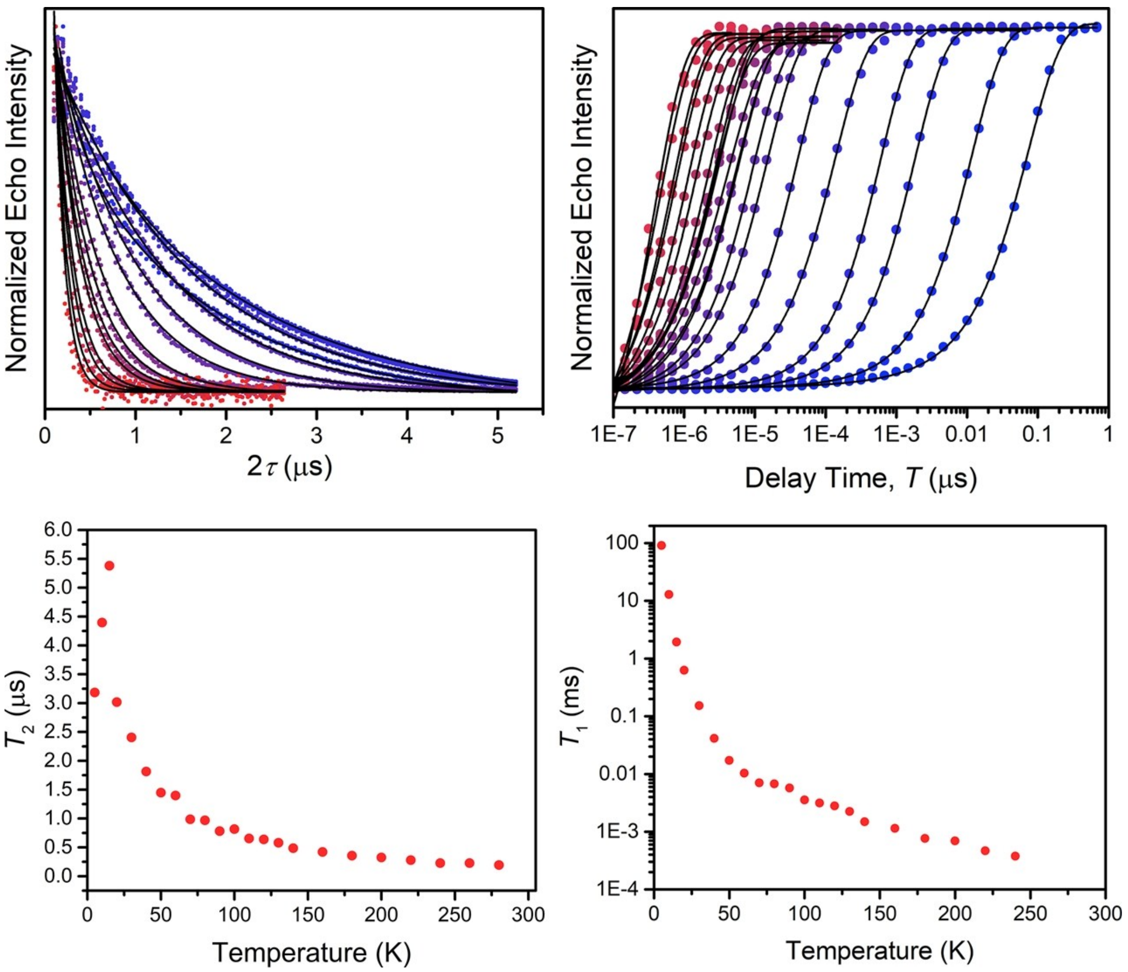

Figure S17 | Pulsed X-band EPR measurements of **4′**. Top-left: variable-temperature T_2 decay curves, collected through application of a Hahn-echo pulse sequence. Black lines are best fits to the data using a mono-exponential decay function. Top-right: saturation recovery curves for **4′** collected through application of twenty consecutive inversion pulses, followed by a Hahn-echo sequence for detection. Black lines are best fits to the data using the decay function described above to account for spectral diffusion. Bottom-left: temperature dependence of T_2 for **4′**. Bottom-right: temperature dependence of T_1 for **4′**. Data were collected from 5 K to 240 K.

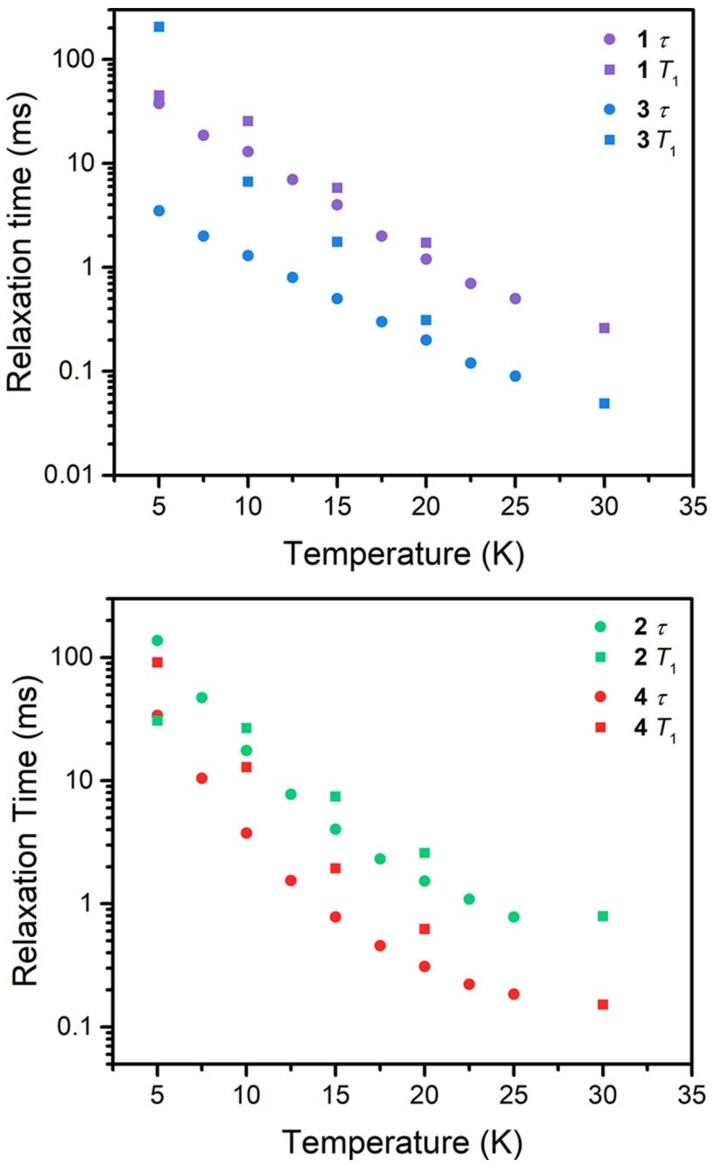

Figure S18 | Overlay of T_1 and τ relaxation times extracted by pulsed EPR and variable temperature ac magnetic susceptibility, respectively, for **1**–**4**. The plots highlight the near overlay of the two parameters, with the greatest divergence observed in **3** owing to cross relaxation.

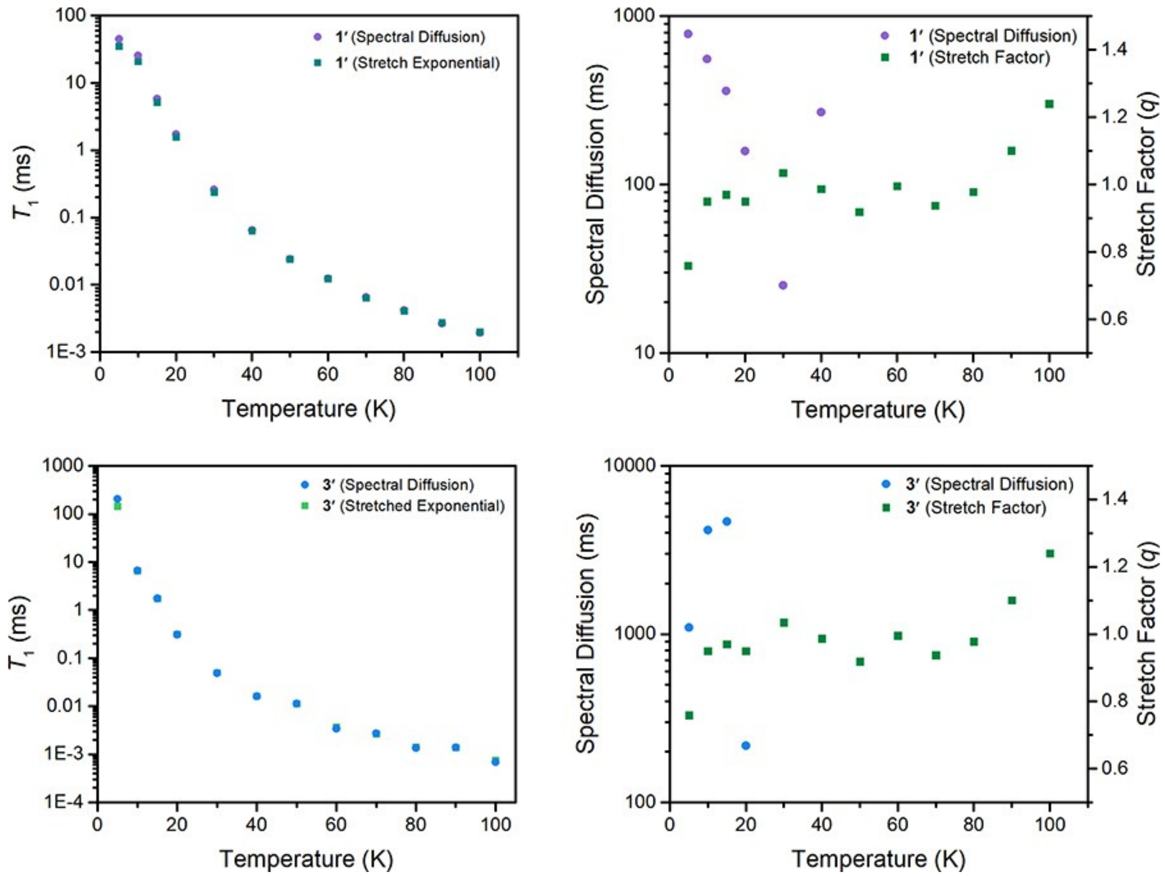

Figure S19 | Left column: Overlay of T_1 data fit using equation 1 above, and a stretched exponential decay function, for **1′** and **3′**, revealing identical fits extracted by both procedures. Notably, the low-temperature data diverges slightly, where spectral diffusion is suspected to be most operative. Right column: Overlay of the spectral diffusion parameter extracted using equation 1 (left axis) with the stretch factor extracted using a stretched exponential fit (right axis). The stretch factor approaches a value of one with increasing temperature as the influence of spectral diffusion becomes more limited and the fits using both procedures coalesce.

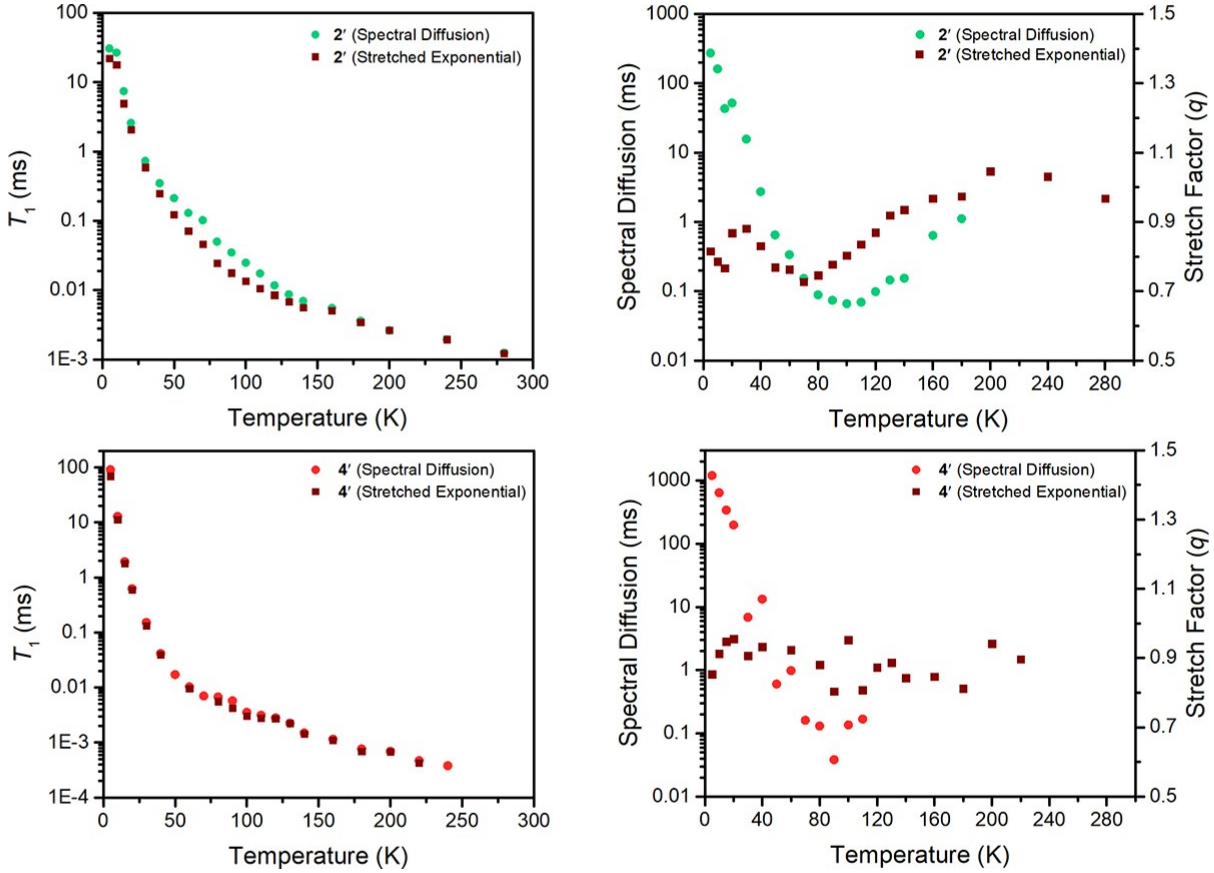

Figure S20 | Left column: Overlay of T_1 data fit using equation 1 above, and a stretched exponential decay curve, for **2′** and **4′**, illustrating very similar fits extracted by both procedures. Right column: Overlay of the spectral diffusion parameter extracted using equation 1 (left axis) with the stretch factor extracted using a stretched exponential fit (right axis). The stretch factor approaches one with increasing temperature as the influence of spectral diffusion becomes more limited and the fits using both procedures coalesce. Notably, spectral diffusion is operative to much higher temperatures in **2′** and **4′** relative to their vanadium(IV) counterparts presented in Figure S19.

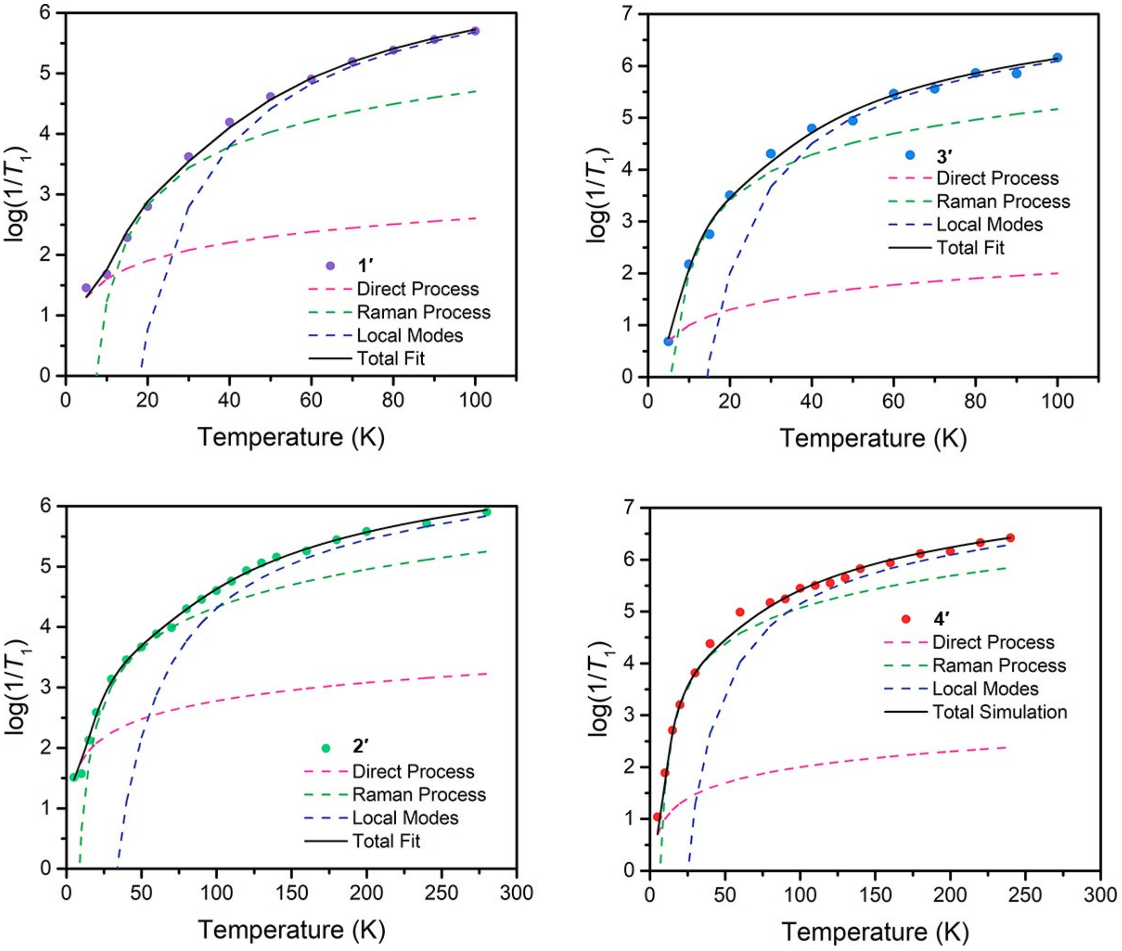

Figure S21 | Fits to the temperature dependence of T_1 to extract the relative contributions of the Direct, Raman, and local modes on spin-lattice relaxation in **1′–4′**. The tabulated fit parameters are presented in Table S22.

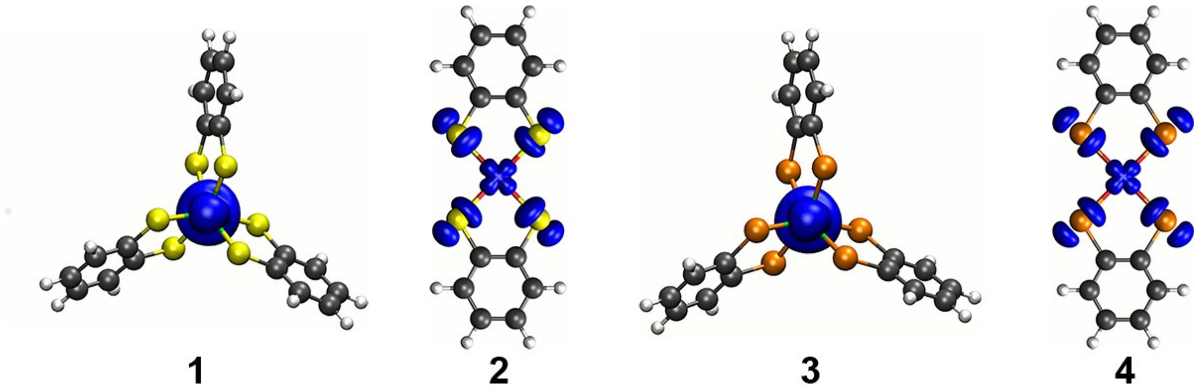

Figure S22 | Plots of spin densities from the M06-L calculation with DMF as the solvent for **1**–**4**.

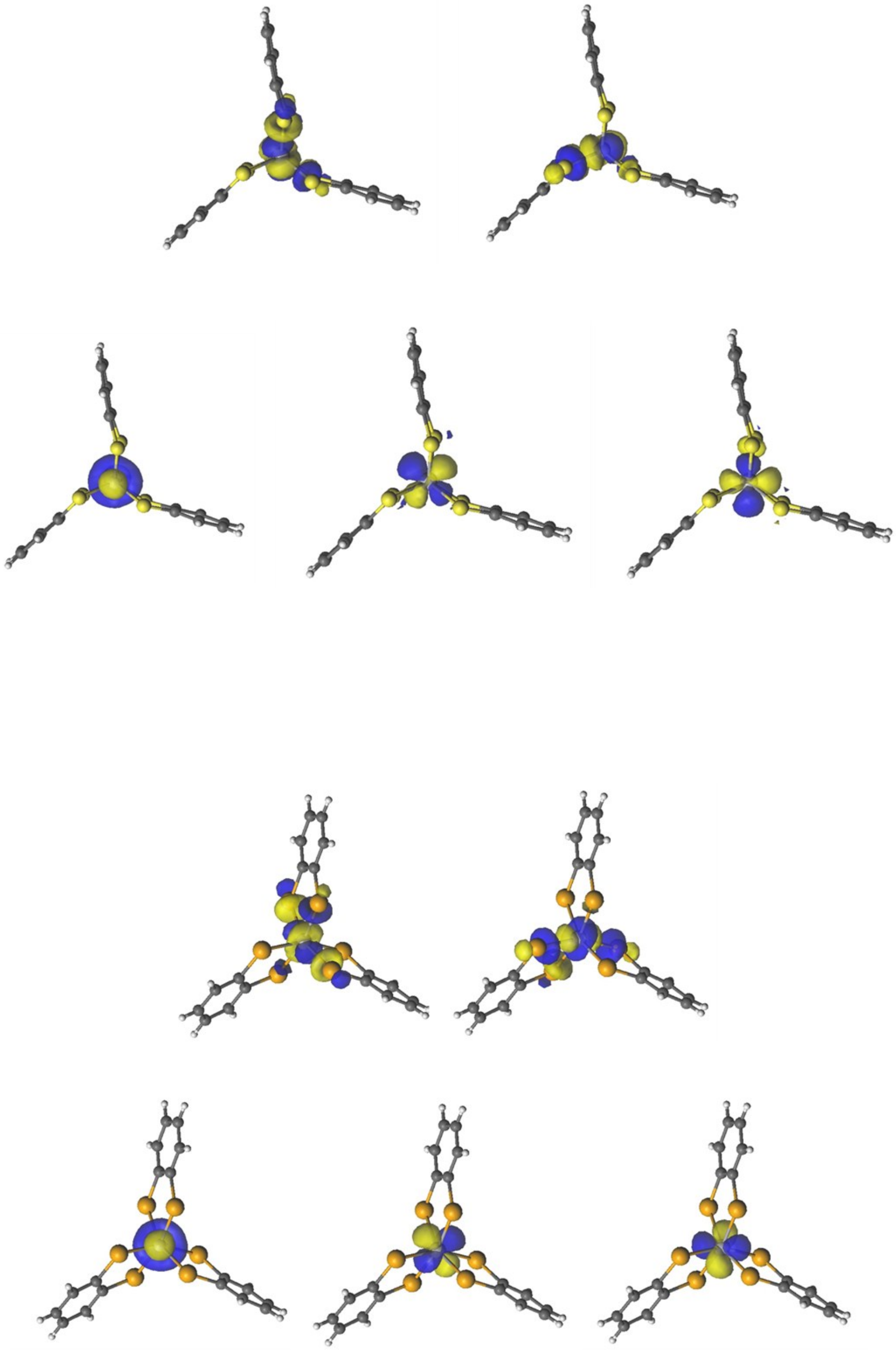

Figure S23 | State-averaged active orbitals from the MS-CASPT2 calculations for **1** (top) and **3** (bottom) with the (1*e*,5*o*) active space.

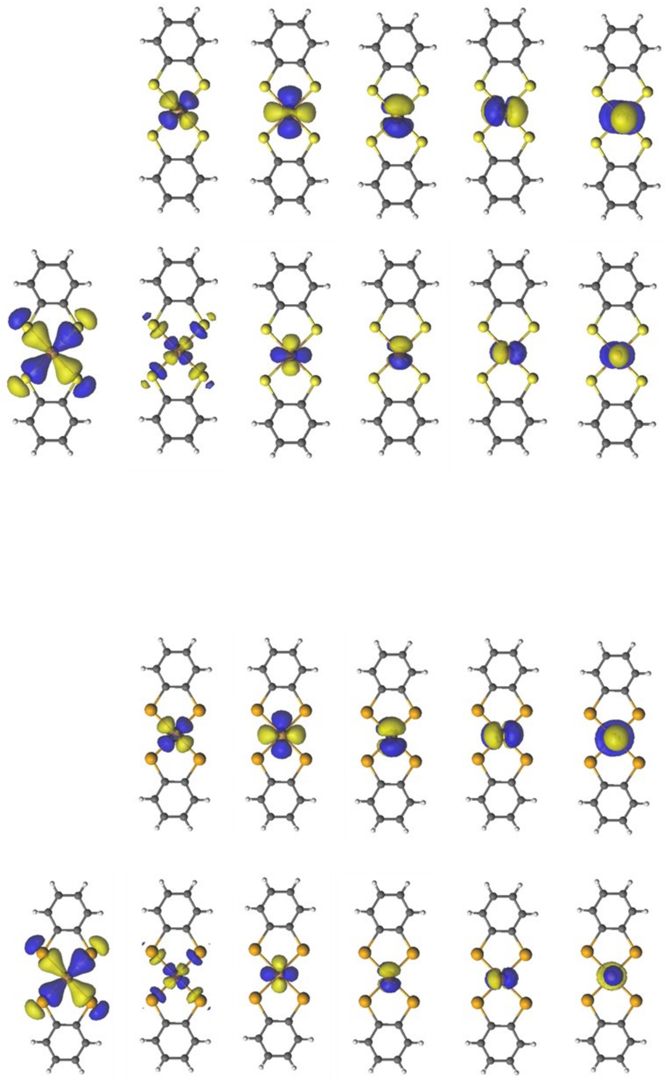

Figure S24 | State-averaged active orbitals from the MS-CASPT2 calculations for **2** (top) and **4** (bottom) with the (11*e*,11*o*) active space.

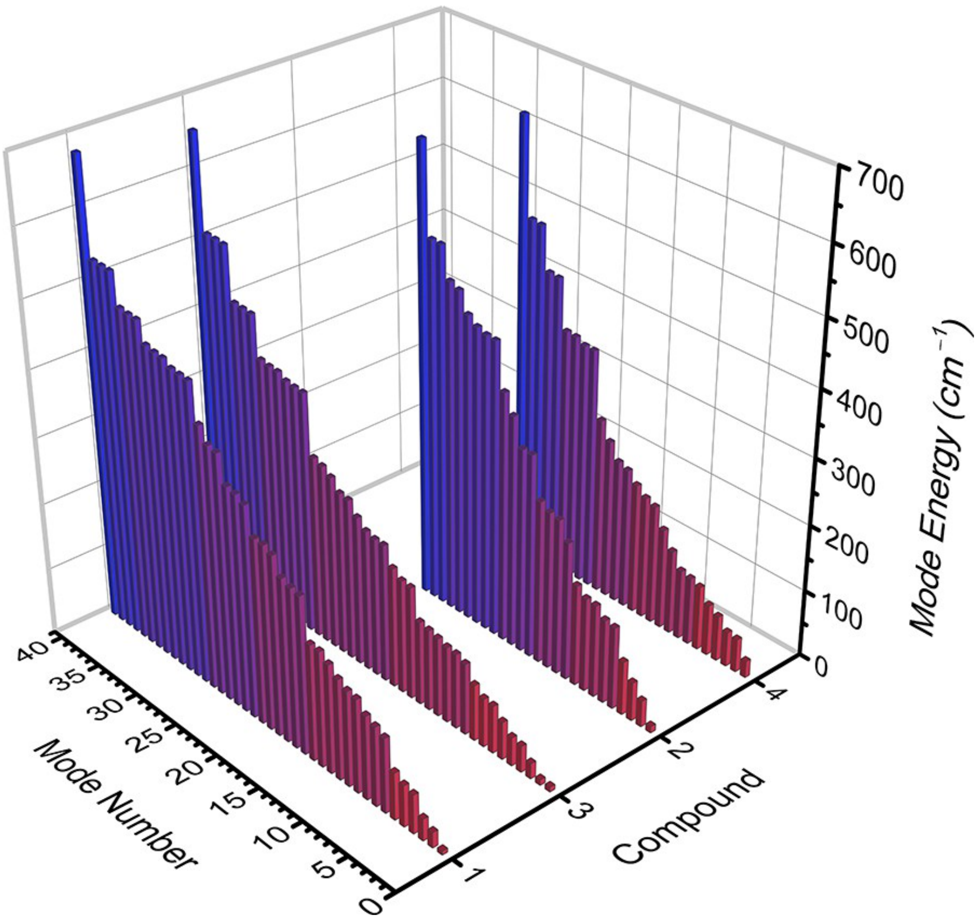

Figure S25 | Computed vibrational modes for compounds **1**–**4** up to 700 cm^{-1}. The copper complexes **2** and **4** feature only 26 vibrational modes in this energy range, while their vanadium counterparts **1** and **3** possess 40 intramolecular vibrational modes within the same energy range. The mode number is only an index for each vibrational mode, and the color scheme bins energy modes in 100 cm^{-1} blocks.

References

(1) Armarego, W. L. F.; Chai, C. *Purification of laboratory chemicals*, 7th ed.; Elsevier Science: Burlington, 2012.

(2) Giolando, D. M.; Kirschbaum, K. *Synthesis*, **1992**, *5*, 451–452.

(3) Theis, B.; Metz, S.; Burschka, C.; Bertermann, R.; Maisch, S.; Tacke, R. *Chem. Eur. J.* **2009**, *15*, 7329–7338.

(4) Rowes, R. A.; Jones, M. M. *Inorg. Synth.*, **1957**, *5*, 113–116.

(5) M. Froneman, D. L. Cheney and T. A. Modro., *Phosphorus Sulfur Silicon Relat. Elem.*, 1990, **47**, 273–282.

(6) Cooper, S. R.; Koh, Y. B.; Raymond, K. N. *J. Am. Chem. Soc.*, **1982**, *104*, 5092–5102.

(7) Xepr; Bruker Biospin, Billerica, MA, United States.

(8) Matlab 12b; The MathWorks, Inc., Natick, MA, United States.

(9) S. Stoll and A. Schweiger, *J. Magn. Reson.*, 2006, **178**, 42–55.

(1 [illegible]

(1 [illegible]

(1 [illegible]

(1 [illegible]

(1 [illegible]

(1 [illegible]

(1 [illegible]

(1 [illegible]

(1 [illegible]

(1 [illegible]

(2 [illegible]

(2 [illegible]

(2 [illegible]

(2 [illegible]

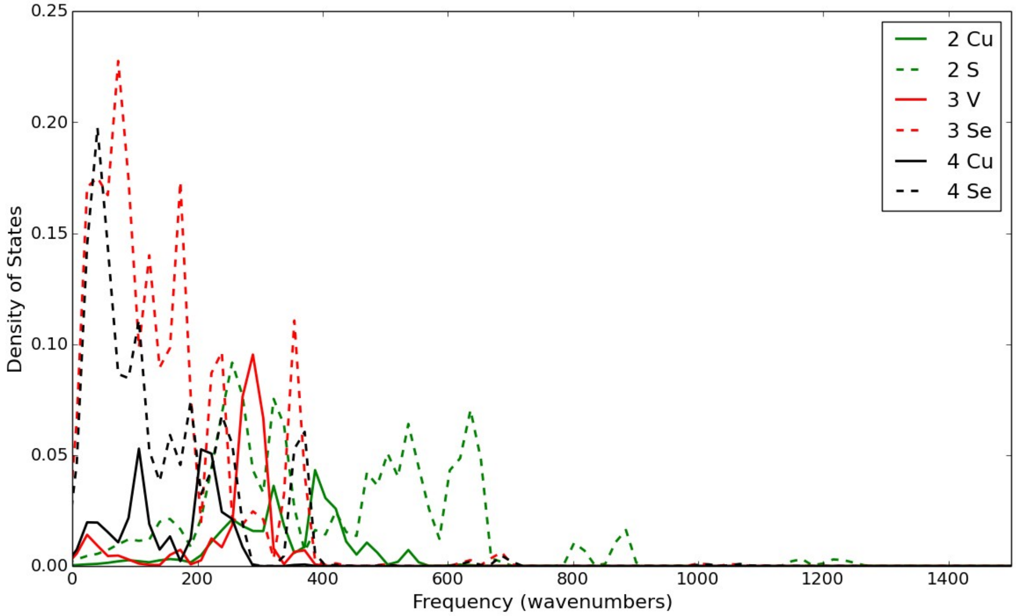

Figure S26 | Computed phonon density of states for **2**–**4**. The phonon density of states are divided into the contributions from the metal center (solid lines) and donor atoms (dashed lines).

(24) A. J. Fielding, S. Fox, G. L. Millhauser, M. Chattopadhyay, P. M. H. Kroneck, G. Fritz, G. R. Eaton and S. S. Eaton, *J. Magn. Reson.*, 2006, **179**, 92–104.

(25) L. Escalera-Moreno, J. J. Baldoví, A. Gaita-Ariño E. Coronado, *Chem. Sci.*, 2018, **9**, 3265–3275

(26) R. D. Shannon, *Acta Cryst. Sec. A* 1976, **32**, 751–767.

(27) M. J. Graham, C.-J. Yu, M. Krzyaniak, M. R. Wasielewski and D. E. Freedman, *J. Am. Chem. Soc.* 2017, **139**, 3196–3201.

(28) M. J. Graham, M. Krzyaniak, M. R. Wasiewlewski and D. E. Freedman, *Inorg. Chem.* 2017, **56**, 8106–8113.
